# Synergistic toxicity in an in vivo model of neurodegeneration through the co-expression of human TDP-43^M337V^ and tau^T175D^ protein

**DOI:** 10.1186/s40478-019-0816-1

**Published:** 2019-11-08

**Authors:** Alexander J. Moszczynski, Madeline Harvey, Niveen Fulcher, Cleusa de Oliveira, Patrick McCunn, Neil Donison, Robert Bartha, Susanne Schmid, Michael J. Strong, Kathryn Volkening

**Affiliations:** 1 Molecular Medicine Research Group, Robarts Research Institute, Schulich School of Medicine and Dentistry, University of Western Ontario, Rm 3246, 100 Perth Dr, London, ON N6A 5K8 Canada; 2 Neuroscience Program, Schulich School of Medicine and Dentistry, University of Western Ontario, London, Canada; 3 Anatomy and Cell Biology, Schulich School of Medicine and Dentistry, University of Western Ontario, London, Canada; 4 Medical Biophysics, Schulich School of Medicine and Dentistry, University of Western Ontario, London, Canada; 5 Imaging Research Group, Robarts Research Institute, Schulich School of Medicine and Dentistry, University of Western Ontario, London, Canada; 6 Clinical Neurological Sciences, Schulich School of Medicine and Dentistry, University of Western Ontario, London, Canada

**Keywords:** Microtubule associated protein tau, TDP-43, Comorbid pathologies, Amyotrophic lateral sclerosis with cognitive impairment, Neurodegeneration, Neuronal cytoplasmic inclusions

## Abstract

Although it has been suggested that the co-expression of multiple pathological proteins associated with neurodegeneration may act synergistically to induce more widespread neuropathology, experimental evidence of this is sparse. We have previously shown that the expression of Thr^175^Asp-tau (tau^T175D^) using somatic gene transfer with a stereotaxically-injected recombinant adeno-associated virus (rAAV9) vector induces tau pathology in rat hippocampus. In this study, we have examined whether the co-expression of human tau^T175D^ with mutant human TDP-43 (TDP-43^M337V^) will act synergistically. Transgenic female Sprague-Dawley rats that inducibly express mutant human TDP-43^M337V^ using the choline acetyltransferase (ChAT) tetracycline response element (TRE) driver with activity modulating tetracycline-controlled transactivator (tTA) were utilized in these studies. Adult rats were injected with GFP-tagged tau protein constructs in a rAAV9 vector through bilateral stereotaxic injection into the hippocampus. Injected tau constructs were: wild-type GFP-tagged 2N4R human tau (tau^WT^; *n* = 8), GFP-tagged tau^T175D^ 2N4R human tau (tau^T175D^, pseudophosphorylated, toxic variant, *n* = 8), and GFP (control, *n* = 8). Six months post-injection, mutant TDP-43^M337V^ expression was induced for 30 days. Behaviour testing identified motor deficits within 3 weeks after TDP-43 expression irrespective of tau expression, though social behaviour and sensorimotor gating remained unchanged. Increased tau pathology was observed in the hippocampus of both tau^WT^ and tau^T175D^ expressing rats and tau^T175D^ pathology was increased in the presence of cholinergic neuronal expression of human TDP-43^M337V^. These data indicate that co-expression of pathological TDP-43 and tau protein exacerbate the pathology associated with either individual protein.

## Introduction

Amyotrophic lateral sclerosis (ALS) is characterized by progressive death of upper and lower motor neurons with death on average 3–5 years after diagnosis [8]. Upwards of 50% of patients are also affected by the presence of cognitive impairment in the form of a florid frontotemporal dementia, or milder changes in cognition and behaviour known as frontotemporal spectrum disorder (ALS-FTSD) [31]. Neurodegenerative diseases, including ALS and the frontotemporal dementias, are characterized by the presence of neuropathological protein inclusions (proteinopathy). The framework of understanding neurodegenerative disease as specific “-opathies” has led to the study of individual proteins mainly in isolation. While it is of critical importance to understand the mechanisms of toxicity of any disease-related protein individually, animal models expressing these single proteins may be inadequate representations of true disease processes as they do not account for the presence of comorbid pathologies. These models constitutively expressed toxic proteins throughout the central nervous system and may have been expressing individual toxic constructs to such a high degree that any subtle effects of the combined expression may have been missed. The concept of “synergistic interactions” between multiple toxic proteins has been suggested previously [4] and cooperative activity of proteins likely have profound effects on the disease state that are, to date, vastly unexplored.

Two of the most frequently observed proteins that comprise pathological inclusions in a broad range of neurodegenerative diseases are TAR-DNA binding protein of 43 kDa (TDP-43) and microtubule associated protein tau (tau). Importantly, TDP-43 is present in motor neuron pathology in nearly all cases of ALS [17], and tau protein pathology has been demonstrated in hippocampal and anterior cingulate brain tissues in the vast majority of cases of ALS with cognitive impairment (ALSci) [32, 37]. In the context of ALSci, while there is evidence that both TDP-43 and tau protein expression are upregulated, they generally do not co-localize within the same cell populations [37]. Beyond ALSci, the coexistence of TDP-43 and tau pathology has been described in the form of concomitant argyrophilic grain disease in ALS [30], Alzheimer’s disease and dementia with Lewy bodies [12].

In this study, we have utilized a rat model of ALSci to investigate the impact of pathological TDP-43 expression on hippocampal tau pathology. We hypothesize that the co-expression of toxic variants of tau and TDP-43 in distinct neuronal populations will result in increased tau pathology and a deterioration of motor function.

## Materials and methods

All experimental protocols were approved by the University of Western Ontario Animal Care Committee (AUP #2013–008 and AUP #2017–108) in accordance with the policies established in the guide to Care and Use of Experimental Animals prepared by the Canadian Council on Animal Care.

### Animals

All rats were provided ad libitum food and water, and were kept on a 12 h:12 h light:dark cycle. Wild-type Sprague-Dawley (SD) rats were acquired from Charles River Canada. ChAT-tTA and TRE-TDP-43^M337V^ breeding adult rats were purchased from the Rat Resource and Research Center (Columbia MO, USA) [14, 41]. The ChAT-tTA line carries the choline acetyltransferase (ChAT) promoter paired with activity modulating tetracycline-controlled transactivator (tTA). The TRE-TDP-43^M337V^ line carries the human TDP-43^M337V^ gene paired with a tTA-dependent tetracycline response element (TRE) driver. Without the presence of the tTA modulator, the TDP-43^M337V^ gene cannot be expressed in this model. When these two lines are crossed, the tTA promoter system activates the TRE-TDP-43^M337V^ transgene, producing an expression pattern that is indistinguishable from constitutive transgene expression [42]. This promoter is silenced by exposure to tetracycline or its derivative doxycycline (DOX; Additional file 1: Figure S1). Therefore, by pairing the tTA and ChAT promoters, constitutive cholinergic neuronal expression of human TDP-43^M337V^ is accomplished. Expression was silenced by the addition of 50 μg/mL DOX in drinking water (from the time of mating to prevent in utero embryonic death) which was replenished every 48 h. To express TDP-43^M337V^ in adult rats, the DOX concentration was reduced by 50% to 25 μg/mL in the drinking water. This reduction was selected in favour of full withdrawal to extend the timeline of expression to avoid the rapid onset of motor dysfunction that has been previously described in these animals [14]. This model was selected for the specificity of cholinergic neuronal expression, which is absent in the hippocampus, but present in lower motor neurons and cortical neurons [2, 5]. After DOX reduction these rats exhibit the loss of motor neurons and a motor phenotype consistent with that seen in human ALS. Genotyping was confirmed by PCR to detect both ChAT and TDP-43. Expression was confirmed by RT-PCR of RNA from animals as previously described [22] and copy number analyses performed to ensure that experimental animals had similar transgene loads [22]. Due to the fact that the lines had to be carried as hemizygous lines, and only females were used in these studies, only 12.5% of the resulting offspring from the crosses were experimental animals.

### Surgical procedure and somatic gene transfer

Somatic gene transfer technique was used to express a recombinant adeno-associated virus (rAAV9) vector, allowing for the specific expression of human tau constructs in the hippocampus [22, 25]. We selected the toxic pseudophosphorylated tau variant human 2N4R tau with amino acid Thr^175^ mutated to Asp to mimic phosphorylation (tau^T175D^) based on our previous studies in which we demonstrated the toxicity of this ALSci associated tau variant in cell culture and rodent models [9, 21, 22, 37]. The constructs were designed to produce an N-terminus GFP-fused tau protein, which was then packaged into rAAV9 virus and injected into the hippocampus of the rats as previously described [22, 25]. Expressed constructs were as follows: GFP-tagged wild-type human 2N4R tau (tau^WT^, *n* = 6), GFP-tagged tau^T175D^ protein (pseudophosphorylated construct; tau^T175D^; *n* = 6), and GFP without tau (GFP; *n* = 6). Vectors, and rAAV9 viruses were produced by Vector Biolabs (Philadelphia PA) and have been reported previously [19, 22].

At 3 months of age, female ChAT-tTA/TRE-TDP-43^M337V^ and wild-type SD rats were inoculated with GFP-tagged tau protein construct-bearing rAAV9 vector through bilateral stereotaxic injection into the hippocampi as previously described [22]. Briefly, animals were anesthetized with isoflurane and head-fixed in a stereotaxic frame with blunt-ended ear bars and a snout mask. After a midline incision in the scalp, four burr holes were drilled into the skull and rAAV9 vectors were injected bilaterally at two sites per hippocampus at the following coordinates (± Bregma): A/P: − 5.5 mm, M/L: ±4.6 mm with D/V: − 3.2 mm, and M/L: ±6.0 with D/V: − 6.0 mm. 3 μl per site was injected over a time of 5 min/injection. The vector volume was adopted from previously reported studies utilizing adenoviral vectors to express tau protein in the hippocampus [22, 25]. Post-surgery, rats were individually housed for 1 week before being returned to paired housing.

### Behavioural testing and analyses

As protein expression changed upon prolonged DOX reduction, ChAT-tTA/TRE-TDP-43M337V (*n* = 18) and TRE-TDP-43^M337V^ (TDP-43 control; *n* = 6) underwent a battery of behavioural paradigms where phenotypic changes between groups were examined. All behavioural testing took place during the light phase (between 7:00 and 19:00 h) and included: open-field test (OFT), prepulse inhibition (PPI) of the acoustic startle response (ASR), sociability behaviour, and CatWalk tests. Animals were handled three to four times prior to commencement of behavioural testing. OFT, PPI and sociability behaviours were assessed prior to 50% DOX reduction (baseline) to determine baseline measures, followed by behavioural testing two and 4 weeks post-DOX reduction. CatWalk testing was also analyzed prior to the DOX reduction, and then at 12 additional time points over the course of the 4 weeks.

#### Open-field testing

OFT was used to determine exploratory locomotor behaviour as cumulative distance traveled and to provide a measure of anxiety-like (thigmotaxis) behaviour. Animals were placed in a square field of 45.7 cm × 45.7 cm dimension and allowed to freely explore for 20 min while the animal head location was monitored by an overhead camera. Data was collected using ANYMAZE software (V4.99, Stoelting, Wood Dale, IL, USA). The total distance traveled as well as the time spent in the center vs. periphery of the box was analyzed across the 20 min testing block.

#### Prepulse inhibition of the acoustic startle response

The acoustic startle response (ASR) was used to measure prepulse inhibition (PPI) which is a behavioural quantification of sensorimotor gating shown to be disrupted in many neurological diseases [28, 33]. PPI reflects an attenuation of the startle response following a loud startling stimulus when it is preceded by a low-intensity stimulus (prepulse) by typically ~ 30-100 ms [16]. PPI is largely assumed to be mediated by mesopontine cholinergic projections that inhibit the startle pathway at the level of the pons, but has been suggested to be modulated by the hippocampus, with the CA1 and dentate gyrus having greatest involvement.

To measure the ASR, as described in Valsamis and Schmid [34], rats were acclimatized to sound-proof startle boxes (Med Associates, VT, USA) with a constant background noise of 65 dB over 3 days for 5 min periods. Input/output function was measured and experimental testing comprised of three blocks: I) Acclimation period for 5 min; II) 20 startle-alone stimuli of 20 ms duration and 105 dB intensity; Block III) 50 pseudorandomized trials of the same startle stimuli paired with or without a preceding prepulse stimulus of 4 ms duration at either 75 or 85 dB intensity with 2 different inter stimulus interval (ISI) of 30 or 100 ms (total of 10 trials/condition). Inter-trial interval was variable between 15 and 25 s. Peak to peak maximum startle magnitude was measured and PPI was expressed as amount of inhibition in percentage of baseline startle: %PPI = [1 - (startle magnitude with prepulse/baseline startle without prepulse)] × 100.

#### Sociability and social recognition testing

Socialization was evaluated due to previous observations of preferential tau expression in this model in the CA2 region of the hippocampus [22]. This expression pattern would not lead to changes in traditional behavioural testing such as the Morris water maze which relies on performant pathway plasticity and not hippocampal CA2. Social memory has been shown to be impaired when this region is pathologically malfunctioning [13]. To test the changes in CA2 function due to pathological tau protein expression, we conducted a three-chamber sociability test, similar to that described by others [6].

In brief, the test apparatus was a rectangular Plexiglas box (40 cm length × 20 cm width × 20 cm height), divided into 3 areas of equal width and separated by perforated Plexiglas. Rats were placed in the middle chamber for 5 min of habituation, after which a familiar animal (littermate) was placed into one of the outer chambers. Test animal was then allowed to investigate both outer chambers and animal activity was recorded using ANYMAZE software (V4.99, Stoelting, Wood Dale, IL, USA) and scored over a 5 min test in which the animal sniffed each compartment (nose within 2 cm of the divider). Animals expressing normal sociability are expected to spend a greater amount of time sniffing the chamber containing the littermate compared to the empty compartment. Behavioural performance was expressed using sociability scores (difference between times spent in stranger vs. empty compartments).

To assess animals for preference for social novelty, an unfamiliar female rat (stranger) was placed in one of the side chambers while a familiar cage mate was placed in the other and test subject was allowed to explore for an additional 5 min. Time spent in each chamber were recorded and a social preference score (difference between time spent in the stranger vs. familiar rat chamber) was calculated for each rat. An animal expressing a normal preference for social recognition is expected to spend more time sniffing the chamber containing the novel stranger.

#### CatWalk testing

All gait assessment was conducted using a CatWalk XT gait analysis system (Noldus Toronto, Canada). Subjects were tested once prior to DOX reduction, followed by testing 3 days/week post-DOX reduction, with each test consisting of 3 consecutive runs/day for 4 weeks. The average of the runs for each week was taken for the calculation of each variable analyzed. Metrics assessed included: body speed and hind paw measures of swing, swing speed, stand, initial contact, and stride length (based on [35]).

### Neuroimaging

Neurite Orientation Dispersion and Density Imaging (NODDI) imaging (dMRI) modality was used for imaging the rats used in this study [20, 39, 40]. Images were acquired at the Centre for Functional and Metabolic Mapping on an Agilent 9.4 Tesla small animal magnetic resonance imaging (MRI) scanner (Santa Clara, CA). Images were acquired using a spin-echo, echo-planar-imaging (EPI) acquisition pulse sequence (4 shots, 2 averages, slice thickness = 500 m, FOV 40x40mm, matrix size 160 × 160, in-plane resolution = 250 × 250 m, TE = 25 ms, TR = 5.0 s) as previously described [20]. A two-shell diffusion sampling scheme was used consisting of 72 b-value = 2000s/mm^2^ directions (gradient strength (G) = 339.1mT/m, time between the start of the first and second diffusion pulse (Δ =14.44 ms, the duration of a single gradient pulse (δ) = 4.32 ms, TE = 25 ms and TR = 5.0 s) and 36 b-value = 1000s/mm2 directions (G = 169.6mT/m, Δ = 14.44 ms, δ = 4.32 ms, TE = 25 ms and TR = 5.0 s). Fifteen b = 0 s/mm^2^ were interspersed throughout the imaging sequence. This imaging sequence has been shown to produce reliable NODDI metrics [20]. Scans were performed after doxycycline reduction when a disease phenotype emerged. The total imaging time was 83 min. Images were pre-processed using fMRI Software Library (FSL, v.5.0.10, Oxford, UK). The NODDI Matlab toolbox (available from the UCL Microstructure Imaging Group) was then used to produce maps of ODI, NDI, and IsoVF.

### Immunohistochemistry

After 30 days of doxycycline reduction, or at end stage as defined in animal use protocols (paralysis or loss of 10% of body mass), rats were intraperitoneally injected with a lethal dose of Euthanyl and trans-cardiacally perfused with heparinized saline (10 units heparin/mL, 0.9% sodium chloride) followed by perfusion fixation with 4% formaldehyde (pH 7.4). Brains were removed and post-fixed in 4% formaldehyde for 24 h before cutting into sections and embedding in paraffin wax. Tissue was then serially sectioned at 4–6 μm and mounted to positively charged microscope slides. With sectioning the brain prior to embedding each slide captured sections from five distinct points along the rostrocaudal axis of the brain per animal.

Immunohistochemistry was conducted to analyze vector expression and the induction of pathology using rabbit anti-GFP (1:750 titer, Life Technologies, Montreal, Canada), mouse anti-human TDP-43 (1:500 titer, Proteintech, Rosemont IL, USA) and mouse anti-PHF tau (AT8; 1:500 titre, ThermoFisher, Montreal, Canada). The characterization of the extent of microglial activation was conducted using rabbit anti-IBA1 (1:1000 titer, Wako, Richmond, VA, USA) while astrocytes were assessed using rabbit anti-GFAP (1:1000 titer, Dako/ Agilent, Santa Clara, CA, USA). Antigen retrieval (10 mM sodium citrate, 0.05% Tween-20 pH 6.0) was conducted for all antibodies using a pressure cooker (2100 Retriever; Aptum Biologics, UK). Endogenous peroxidase was quenched with 3% hydrogen peroxide (VWR, Mississauga, Canada). Primary antibody incubation was performed at 4 °C overnight in blocking buffer (5% BSA, 0.3% Triton-X 100 in 1X PBS). After washing, secondary antibody (1:200 titer biotinylated IgG) incubation was performed for 1 h at room temperature in blocking buffer. Antigen:antibody complex was visualized with the horseradish peroxidase coupled Vectastain ABC kit (Vector Laboratories CA, USA) according to the manufacturer’s instructions, followed by substrate development with DAB. Counterstaining was performed using Harris’ haematoxylin.

To co-stain for axon integrity and myelination, a modified SMI-31/Luxol fast blue immunohistochemistry was performed (SMI-31:10,000 titer, BioLegend, San Diago, USA) as described in Moszczynski et al. (Applied Immunohistochemistry and Molecular Morphology, in press). Briefly, Luxol fast blue staining was performed after SMI-31 IHC by staining in 1% Luxol fast blue ((Sigma, St. Louis, MO, USA) overnight at 56 °C after slides were dehydrated post-DAB development. Luxol fast blue differentiation was performed with 0.05% lithium carbonate and 70% ethanol, followed by dehydration to xylenes and converslipping.

### Co-localization and fluorescence staining

To visualize multiple proteins, TDP-43 was probed with mouse anti-human TDP-43 (1:500 titer, Proteintech) in conjunction with rabbit anti-cleaved caspase-3 (Asp175; clone 5A1E; 1:200 titer, Cell signaling, MA, USA), and mouse anti-GFP (1:250 titer, Abcam) in conjunction with rabbit anti-IBA1 (1:1000 titer, Wako) or rabbit anti-GFAP (1:1000 titer, Dako) primary antibodies overnight at 4 °C and Alexafluor goat anti-mouse 488, donkey anti-rabbit 488, and goat anti-rabbit 555 or donkey anti-mouse 565 secondary antibodies (all used at 1:200 titer, ThermoFisher) for 1 h at room temperature. Slides were visualized within 24 h of labeling by confocal imaging on a Zeiss LSM 510 Meta multiphoton confocal microscope or on an EVOS M7000 imaging system (ThermoFischer).

### Quantification and statistical analysis

#### GFP-tau pathology scoring

Photomicrographs of all GFP-expressing hippocampal regions were taken for 3 rats in each group (GFP, tau^WT^, tau^T175D^) on both ChAT-tTA/TRE-TDP-43^M337V^ (TDP-43 expressing) and wild-type SD background strain using the 20x objective on an Olympus BX45 light microscope. Pathology was scored by a trained observer who was blinded to the expression group. The total number of pathological events was counted for each field.

#### Motor neuron counts

Lumbar ventral horns were photographed for 3 rats from each tau construct expressing group in the TDP-43 expressing ChAT-tTA/TRE-TDP-43^M337V^ line using the 10x objective of an Olympus BX45 light microscope. After image acquisition, the total number of motor neurons present (identified by size and the presence of a prominent nucleus with a typical ‘clock face’ appearance of the chromatin) in the ventral horn of all lumbar spinal cord sections was counted by a blinded observer.

#### TDP-43 pathology scoring

After staining for human TDP-43, lumbar ventral horns for 3 rats from each tau construct expressing group on the ChAT-tTA/TRE-TDP-43^M337V^ line were photographed using the 10x objective of an Olympus BX45 light microscope. Pathology, defined as the presence of at least one skein or aggregate, was quantified by a blinded observer and then the number of pathology-bearing motor neurons was normalized against total number of motor neurons in each ventral horn (pathology-bearing motor neurons/total motor neurons).

#### Glial activation quantification

After staining with IBA1 or GFAP antibodies, images were acquired using the 20x objective of an Olympus BX45 light microscope. A minimum of 5 images were acquired spanning the entire hippocampus in each of 3 animals per group. Images were then quantified using ImageJ to generate a numerical percentage of the total field of view that was stained positive.

#### Behavioural assessments

IBM SPSS Statistics 23.0 was used for statistical analysis. Data were expressed as group means ± the standard error of the mean (SEM). A two-way mixed-design analysis of variance (ANOVA) was used to analyze the majority of the data, with “time” or “DOX reduction” (3 levels: Before DOX reduction (baseline) vs. 2 weeks after DOX reduction vs. 4 weeks after DOX reduction) as the within-subject factor and “genotype” (2 levels: ChAT-tTA/TRE-TDP-43M337V vs. TRE-TDP-43^M337V^) or “tau” (3 levels: GFP vs. tau^WT^ vs. tau^T175D^) as the between-subject factors. All behavioural tests, other than CatWalk, considered the tau injection groups during analyses. Normality was assessed using a Shapiro-Wilk test for normality. Homogeneity of variance was assessed using Levene’s test of homogeneity. Homogeneity of covariance was assessed using Box’s M test. Mauchly’s test of sphericity was used to test the assumption of sphericity for two-way interactions. If sphericity was violated, corrections were applied based on the ε value and the Huynh-Feldt correction. Significance was accepted when *p <* 0.05 and outliers were removed by box plot analysis. We determined the average %PPI for each prepulse type (dB and ISI combinations) and performed two-way ANOVA (prepulse type × genotype) and (prepulse type × tau). Since there were no significant main interaction on PPI, all PPI ISI and prepulse groups were re-assessed together and graphed accordingly. For all statistically significant behavioural results where post hoc analyses were appropriate, a Student’s t test with Bonferroni corrections was performed.

#### Statistical analysis of histopathological data

Statistical analyses were conducted using SigmaPlot 10.0 software. A one-way analysis of variance (ANOVA) was conducted following a Shapiro-Wilk test for normality. Post-hoc Tukey’s test was conducted and a *p* ≤ 0.05 was considered significant. For non-normal data, a Kruskal-Wallace one-way ANOVA on ranks was conducted followed by a Dunn’s pairwise test for multiple comparisons.

#### Neuroimaging

A Multivariate Analysis of Variance was performed to detect any statistically significant differences between mean ODI, NDI, and IsoVF within the corpus collosum and hippocampus for each group (GFP, tau^WT^, and tau^T175D^).

## Results

### ChAT-tTA/TRE-TDP-43M^337V^ animals express human TDP-43, develop a marked motor phenotype, and exhibit loss of motor neurons upon withdrawal of doxycycline

**Fig. 1:**
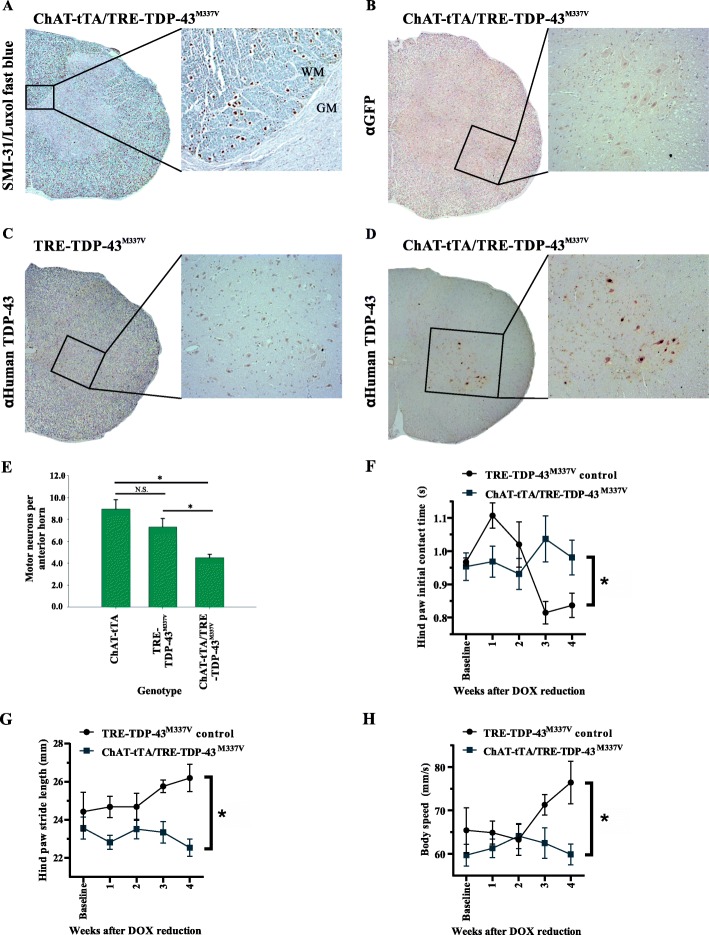
Expression of TDP-43^M337V^ affects motor phenotype. **a** Immunohistochemical staining for phosphorylated high molecular weight neurofilament (SMI-31) counterstained with Luxol fast blue shows no apparent loss of myelin in the corticospinal tracts. Inset = higher magnification of the corticospinal tract showing axonal cross sections (brown) and myelin (green). WM = white matter, GM = grey matter. **b** GFP is not expressed in the rat spinal cord when not fused to tau. **c** Human TDP-43 is not expressed in the TRE-TDP-43^M337V^ rat spinal cord without the ChAT-tTA transgene. **d** Human TDP-43 is expressed in cholinergic motor neurons in the spinal cord of ChAT-tTA/TRE-TDP-43^M337V^ rats (brown staining). All photomicrographs represent rats at 30 days post doxycycline (DOX) reduction. All images were taken at 20x, insets at 40x. **e** ChAT-tTA/TRE-TDP-43^M337V^ rats exhibit a reduced number of motor neurons in lumbar spinal cord compared to non-expressing parental transgenic lines (*n* = 3/group, 30 days after DOX reduction, mean ± SEM). * = significant Dunn’s post-hoc test for multiple comparisons (*p* < 0.05) following a one-way ANOVA (*p* = 0.01). **f** Mixed ANOVA found a statistically significant interaction between the genotype (ChAT-tTA/TRE-TDP-43^M337V^) and DOX reduction time on hind paw initial contact time in CatWalk XT gait analysis (*p = 0.010; *n* ≥ 4/group), suggesting that TDP-43 expressing rats took longer to initially contact the walkway with 50% DOX reduction over time, compared to TDP-43 non-expressing controls. **g** There was a statistically significant interaction between the TDP-43^M337V^ and DOX reduction time on hind paw stride length (*n* ≥ 6/group, *p* = 0.024). However, there was no significant main effect of time, suggesting that DOX withdrawn TDP-43 expressing animals generally took smaller strides over time, compared to TDP-43 non-expressing controls. **h** There was a statistically significant interaction between the TDP-43^M337V^ and DOX reduction time on body speed (*n* ≥ 6/group, *p =* 0.039) but no main effect of time, suggesting that DOX reduced TDP-43 expressing rats generally slowed down over time, compared to controls

After 30 days of 50% DOX reduction in drinking water, all rats on the genetic background containing ChAT-tTA/TRE-TDP-43^M337V^ developed a hind-limb motor phenotype consistent with that previously described [41]. Hind limb muscle wasting was observed along with weakness resulting in an inability to lift the pelvis off the ground and dragging of the hindlimbs. This was evident by motor impairment as early as week 3 using the CatWalk gait analyses which demonstrated gait disturbances, including increased latency of hind paw initial contact, reduced stride length and decreased body speed (Fig. 1f-h).

**Fig. 2:**
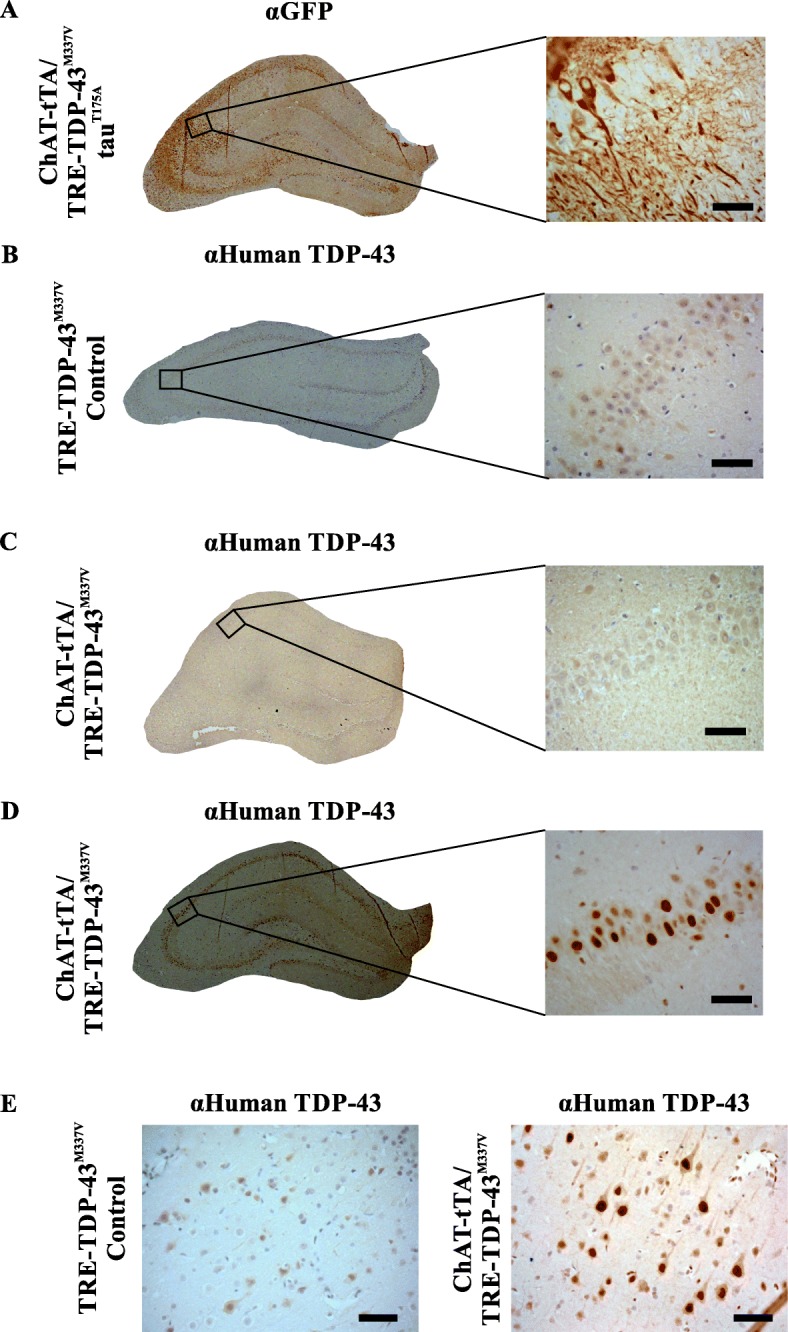
Cerebral expression of human GFP-tagged tau protein constructs and human TDP-43^M337V^. **a** Composite image of hippocampus from ChAT-tTA/TRE-TDP-43 rat expressing tau^T175D^ probed for GFP shows expression of GFP-tagged tau^T175D^ protein throughout the hippocampus with prominent neuronal and neuritic expression in the CA2 region (brown). This same expression pattern was observed for all three AAV9 injected groups (GFP, GFP-tagged tau^WT^, and GFP-tagged tau^T175D^; *n* = 3 for each group). **b** TRE-TDP-43^M337V^ hippocampus probed for human TDP-43 reveals no expression of human TDP-43 confirming that TDP-43 is not expressed without the ChAT-tTA transgene (*n* = 3). **c** and **d** ChAT-tTA/TRE-TDP-43^M337V^ transgenic rat hippocampus probed for human TDP-43 showed a variety of expression during DOX withdrawal, ranging from extremely low levels (**c**) to robust TDP-43 expression (**d**). **e** TRE-TDP-43^M337V^ rat cortex probed for human TDP-43 reveals no evidence of expression (left) while ChAT-tTA/TRE-TDP-43^M337V^ rat cortex shows human TDP-43 expression that is predominantly nuclear (right). All composite images taken with a 20x objective. Scale bar = 10 μm

**Fig. 3:**
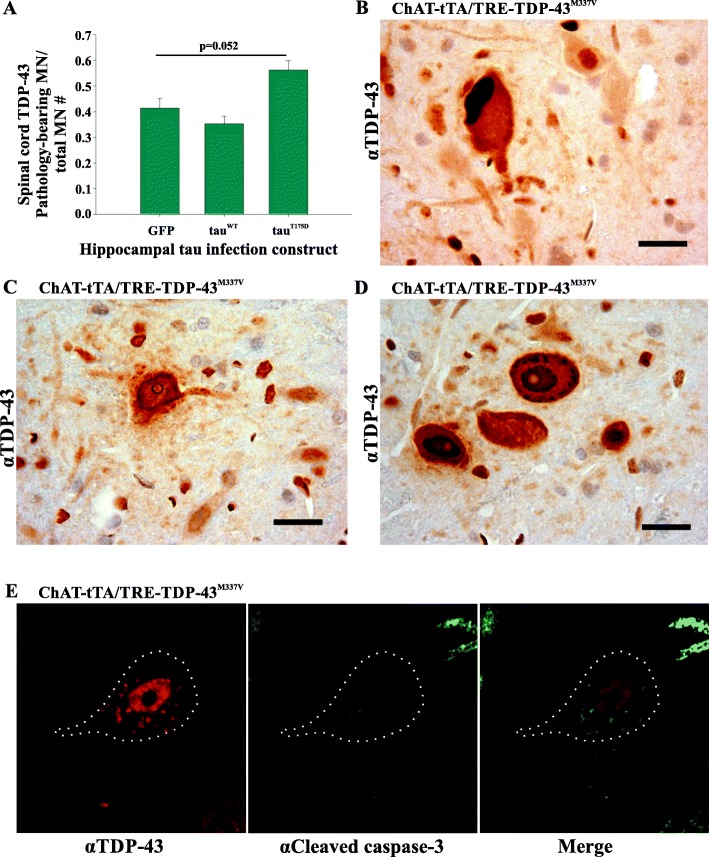
Spinal cord TDP-43 pathology in ChAT-tTA/TRE-TDP-43^M337V^ rats was detected by immunohistochemistry with anti-TDP-43 antibodies regardless of hippocampal injected tau construct. **a** Frequency of pathology-bearing motor neurons normalized against total number of motor neurons. The presence of spinal TDP-43 pathology approaches significance in the rats expressing tau^T175D^ when compared to GFP expressing rats (*p* < 0.1). MN = motor neuron, GFP = GFP only control, tau^WT^ = GFP-tagged tau^WT^ protein construct, tau^T175D^ = GFP-tagged tau^T175D^ protein construct). This was not significant by one-way ANOVA (*p* = 0.052). **b** Photomicrograph showing spinal cord dorsal horn TDP-43 cytosolic inclusion in a motor neuron. **c** Photomicrograph of a spinal motor neuron showing depleted nuclear TDP-43 with cytosolic TDP-43 and surrounding glial cells. **d** Photomicrograph of spinal motor neurons exhibiting punctate TDP-43 pathology. **e** Confocal imaging detecting cleaved caspase-3 (green) in a motor neuron co-expressing human TDP-43 (red) punctate pathology. This was observed in all ChAT-tTA/TRE-TDP-43^M337V^ rats where TDP-43^M337V^ displayed pathology regardless of hippocampal tau expression (*n* = 3 for all groups). Scale bar = 20 μm

In the ChAT-tTA/TRE-TDP-43^M337V^ rats human TDP-43 expression was observed at 30 days post DOX reduction in both ventral horn motor neurons (Fig. 1d) and cortical neurons (Fig. 2d) consistent with previous studies [14]. Expression of human TDP-43 was not observed in wild-type SD (data not shown), ChAT-tTA (data not shown), or TRE-TDP-43^M337V^ (without ChAT-tTA transgenes) groups (see Fig. 1c, and Fig. 2b, e). The hippocampal expression of human TDP-43 on DOX withdrawal was variable in the ChAT-tTA /TRE-TDP-43^M337V^ rats, varying from extremely weak to strong expression (compare Fig. 2c, d). When examined for human TDP-43 expression in spinal motor neurons, all ChAT-tTA/TRE-TDP-43^M337V^ rats demonstrated TDP-43 pathology consistent with that observed in human ALS including nuclear cytoplasmic inclusions (Fig. 3b), nuclear depletion of TDP-43 (Fig. 3c), and punctate nuclear TDP-43 deposition (Fig. 3d). Also consistent with previous results in this model [41], human TDP-43^M337V^ expression in spinal motor neurons led to the identifiable presence of pro-apoptotic markers including caspase-3 cleavage and activation in motor neurons (Fig. 3e).

To determine if loss of motor neurons was occurring as previously described, motor neurons in the ventral horn of spinal cords were counted across a minimum of 4 separate whole lumbar spinal cord sections in 3 animals per group. Motor neurons were identified by their large size and classic “clock-face” nuclear staining with H&E stain. As previously described in this model [14], mutant ChAT-tTA/TRE-TDP-43^M337V^ rat (TDP-43 expressing) lumbar spinal cord ventral horns contained a significantly reduced number of motor neurons (Mean ± SEM; 4.5 ± 0.27) compared to either ChAT-tTA (8.94 ± 0.87) or TRE-TDP-43^M337V^ (7.3 ± 0.63) non-expressing parental strains after significant one-way ANOVA on ranks (*p* < 0.001) followed by Dunn’s post-hoc analysis for multiple comparisons (*p* < 0.05 for each comparison; Fig. 1e). However, contrary to previous observations [14], no evidence of degeneration of myelin or axons in the corticospinal tract was observed. Immunohistochemistry detecting phosphorylated high molecular weight neurofilament (SMI-31 antibody), coupled with Luxol fast blue stain, showed preservation of the corticospinal tracts (Fig. 1a), which may be related to the fact that our withdrawal scheme for DOX was staged and not abrupt; however, the reasons behind this are not known at this point.

### Hippocampal injection of tau into rats on the ChAT-tTA/TRE-TDP-43^M337V^ background

Rats on the ChAT-tTA/TRE-TDP-43^M337V^ genetic background were injected into the hippocampus with AAV9 virus carrying constructs to express GFP alone (control), GFP fused to tau^WT^, or GFP fused to tau^T175D^. The expression of human TDP-43 and tau was then examined. The tau^T175D^ mutation was used to mimic phosphorylation of the threonine residue at amino acid 175 in human tau. Phosphorylation at this site is pathological as has been determined in previous work [1, 9, 21, 22, 24] and is expressed in humans with ALS and cognitive impairment. The aim of this study was to examine if tau and TDP-43, while expressed in different cell populations, may alter spinal cord or hippocampal pathology when co-expressed.

In spinal motor neurons, all ChAT-tTA/TRE-TDP-43^M337V^ rats (with and without hippocampal injection of any of the GFP/GFP-tau constructs) demonstrated TDP-43 pathology consistent with that observed in human ALS. TDP-43 pathology was quantified by counting all visible neurons (as assessed by size, position in the ventral horn and nuclear morphology) and assigning a binary response where the presence of an aggregate, skein or punctate staining (for examples see Fig. 3b, d) was positive, and the absence of all of these was considered negative. The simple redistribution of TDP-43 from the nucleus or a clearing of the nucleus was not considered pathological for this study (Fig. 3c) to avoid potential over-estimation of the pathology. The number of cells displaying aggregates or skeins were normalized against the total number of motor neurons in each ventral horn analyzed. One-way ANOVA revealed no significant difference in the extent of pathology between groups (positive motor neurons/total motor neurons; mean ± SEM; GFP = 0.41 ± 0.09, tau^WT^ = 0.35 ± 0.06, tau^T175D^ = 0.59 ± 0.03, ANOVA *p* = 0.052). It is noteworthy that a trend approaching significance (*p* < 0.1) was observed in the tau^T175D^ expressing group for increased motor neuron TDP-43 pathology (Fig. 3a). It would be of interest to examine this further in the future to determine if significance is ultimately reached with a larger number of animals, or by stratifying the animals based on either time to end stage after doxycycline withdrawal, or by TDP-43 expression level in the brain after DOX withdrawal.

All AAV9 injected rats expressed GFP specifically in the hippocampus as previously described [22], with a higher level of expression noted in the CA2 region extending from the neuronal cell bodies into the axonal projections (Fig. 2a). Tau^WT^ and tau^T175D^ were expressed as fusion proteins with N-terminally positions GFP, so that detection with the anti-GFP antibody would necessarily detect tau.

Caspase-3 cleavage was also examined in ChAT-tTA/TRE-TDP-43^M337V^ rats. TDP-43 pathology was detected in all TDP-43 expressing spinal cords, regardless of which tau (or GFP) was injected, suggesting that the loss of motor function was due to the loss of motor neurons through a caspase-dependent cascade leading to cell death. These observations were consistent with those previously published [41].

### Tau pathology in rats

**Fig. 4:**
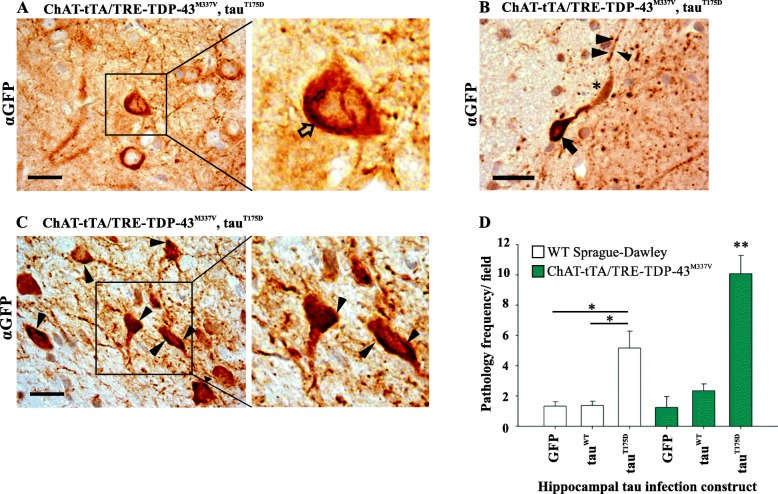
GFP-tau pathology is increased in the presence of TDP-43^M337V^ expression. **a** GFP-tau positive hippocampal neuron demonstrating a fibril type inclusion (inset, open arrow). **b** Photomicrograph of a GFP-tau immunoreactive hippocampal neuron demonstrating a neuronal inclusion (arrow), axonal swelling (asterisk), and axonal beading (arrowheads). Note that the latter is nonspecific as it was observed across all groups. **c** GFP-tau positive hippocampal neurons showing frequent incidence of pathological inclusions (arrowheads, inset). Scale bars = 20 μm. **d** Bar graph showing frequency of pathological inclusions in hippocampus at 20x magnification in each group (GFP = GFP only control, tau^WT^ = GFP-tagged tau^WT^, tau^T175D^ = GFP-tagged tau^T175D^). White bars indicate wild-type Sprague-Dawley rats injected with rAAV9 GFP-tau constructs, green bars indicate ChAT-tTA/TRE-TDP-43^M337V^ rats expressing human TDP-43^M337V^ injected with rAAV9 GFP-tau constructs (*n* = 3 animals per group)

The constructs to express tau were designed specifically to be fusions with GFP so that the anti-GFP antibody could be used to detect tau. GFP-tau immunoreactive neuronal cytoplasmic inclusions were observed in the tau^T175D^ expressing rats in all hippocampal regions expressing GFP constructs. In the tau^T175D^ infected cells both fibrils and tangles were easily detected (see Fig. 4a, b, and c for examples as observed using anti-GFP immunohistochemistry). Consistent with previous results [22] we observed axonal beading in all groups within GFP-immunoreactive neuritic processes, suggesting that this was a nonspecific feature of the invasiveness of the surgery itself (Fig. 4b, arrowheads). The frequency of neuropathology was significantly increased in the tau^T175D^ on wild-type Sprague-Dawley background (Mean ± SEM; GFP = 1.32 ± 0.28, tau^WT^ = 1.36 ± 0.28, tau^T175D^ = 5.15 ± 1.12; Fig. 4d). This was further elevated in the human ChAT-tTA/TRE-TDP-43^M337V^ rats where tau^T175D^ pathology was nearly doubled compared to its wild-type SD background counterpart (GFP expressing ChAT-tTA/TRE-TDP-43^M337V^ 1.25 ± 0.71, tau^WT^ expressing ChAT-tTA/TRE-TDP-43^M337V^ 2.34 ± 0.46, tau^T175D^ expressing ChAT-tTA/TRE-TDP-43^M337V^ 10.08 ± 1.20). The difference in pathology between the two rAAV9 tau^T175D^ expressing groups was significant (*p* = 0.004) while the presence of tau pathology in the ChAT-tTA/TRE-TDP-43^M337V^ group was significantly elevated relative to all other groups (*p* < 0.05).

**Fig. 5:**
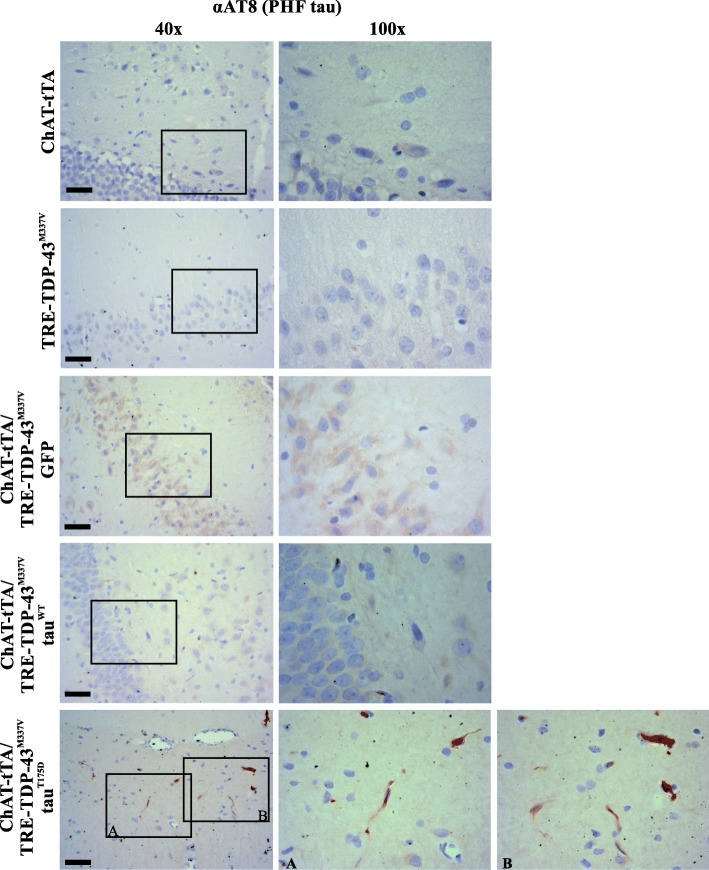
Hippocampal expression of human GFP-tagged tau protein using AT8 antibody against paired helical filament tau in parental lines and rats expressing TDP-43 with GFP and/or tau expression. Representative sections from the CA1 region of the hippocampus in rats. Parental lines (ChAT-tTA and TRE-TDP-43^M337V^) were not injected with any rAAV9 (top two rows). Rats expressing TDP-43 (ChAT-tTA/TRE-TDP-43^M337V^ were injected with either rAAV9 encoding GFP alone (GFP), or GFP-fused wildtype tau (tau^WT^) or GFP-fused tau^T175D^ (tau^T175D^). Note the staining in the ChAT-tTA/TRE-TDP-43^M337V^ rats expressing tau^T175D^ (brown, bottom row; *n* = 3 for each group). Photos in the left column were taken with 40X objective, right column with oil immersion 100X objective. Scale bar = 20 μm

To confirm that the pathology seen with the anti-GFP antibodies reflects the depostion of human tau protein, rat hippocampi were probed with the AT8 antibody to paired helical filaments of tau (Fig. 5). No PHF immunoreactivity was detected in either parental strain (ChAT-tTA or TRE-TDP-43^M337V^) or with the expression of TDP-43 (ChAT-tTA/TRE-TDP-43^M337V^) with injection of GFP or GFP-tau^WT^-rAAV9. PHF tau was robustly detected within neurons in the ChAT-tTA/TRE-TDP-43^M337V^ expressing rats that also expressed tau^T175D^ (Fig. 5). When taken together with the GFP staining described above, it is clear that tau pathology is increased in the hippocampus of rats expressing TDP-43^M337V^.

**Fig. 6:**
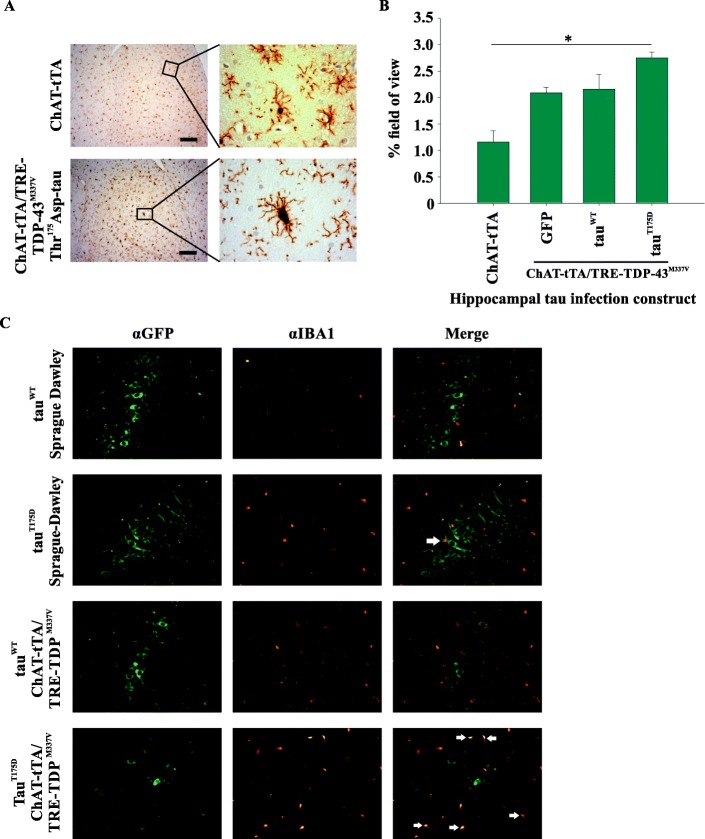
Microgliosis is increased in the hippocampus of ChAT-tTA/TRE-TDP-43^M337V^ rats co-expressing pathogenic human tau^T175D^ protein. **a** Representative photomicrographs showing IBA1 stained microglia in ChAT-tTA control and tau^T175D^ expressing ChAT-tTA/TRE-TDP-43^M337V^ rat hippocampus. Insets show an activated microglial cell with enlarged cell body in ChAT-tTA/TRE-TDP-43^M337V^ transgenic rat brain and resting microglial cell in ChAT-tTA control. Light microscopy low magnification images were taken with 20x objective, insets taken with 100x oil immersion objective. Scale bar = 50 μm. **b** Quantification of IBA1 staining across the field of view shows increased coverage (proxy for microglial activation) in ChAT-tTA/TRE-TDP-43^M337V^ rats with human tau^T175D^ expressed in the hippocampus. All quantification represents either the ChAT-tTA control group (non-expressing for TDP-43^M337V^ or any GFP construct) or hippocampal GFP-tau expressing groups on ChAT-tTA/TRE-TDP-43^M337V^ transgenic background (GFP = green fluorescent protein, tau^WT^ = GFP-tagged tau^WT^, tau^T175D^ = GFP-tagged tau^T175D^ human tau). *p < 0.05 between indicated groups. **c** Fluorescence microscopy of the hippocampus of wild type rats (no ChAT/no TDP-43 transgenes; top two rows) and rats expressing human TDP-43 (bottom two rows) with tau^WT^ (top row and third row) or tau^T175D^ (second and four rows) co-probed with antibodies to GFP (fusion protein with tau) and IBA1. White arrows indicate IBA1 positive microglial cells in close-proximity to GFP-positive (tau positive) hippocampal neurons. Three animals were examined for each group and photomicrographs shown are representative for each group

IBA1 staining revealed activated microglia in Tau^T175D^ expressing ChAT-tTA/TRE-TDP-43^M337V^ rat hippocampi that was not observed in ChAT-tTA control rats (Fig. 6a). An analysis of the percent area coverage of the field (a marker of microglial activation) revealed a higher overall amount of positive staining in ChAT-tTA/TRE-TDP-43^M337V^ rats expressing tau^T175D^ relative to the ChAT-tTA parental control strain (*p* < 0.05) while GFP and tau^WT^ groups were not statistically significant from the parental control strain (Fig. 6b). Fluorescent staining revealed a phenotype of IBA1 positive microglia in close proximity to tau^T175D^ positive hippocampal neurons, but not in other groups (Fig. 6c). Although no significant differences in GFAP staining were observed between hippocampal construct expressing groups within ChAT-tTA/TRE-TDP-43^M337V^, a trend toward increased GFAP-positive staining was observed in tau^T175D^ hippocampus (Additional file 2: Figure S2, ANOVA *p* = 0.072).

### Behaviour testing in TDP-43 expressing rats confirm motor deficits while the addition of tau^T175D^ expression in these rats leads to subtle behaviour changes

In CatWalk gait analyses, mixed ANOVA detected a significant interaction between the TDP-43^M337V^ expression and DOX reduction time on hind paw initial contact time, (F (_4, 72_) = 3.572, *p =* 0.010; Fig. 1f), with no main effect of time, (F (_4, 72_) = 1.528, *p =* 0.203) suggesting that ChAT-tTA/TRE-TDP-43^M337V^ animals took longer to initiate hind paw contact with DOX reduction over time. No effect of genotype was found, (F (_1, 18_) = 0.095, *p =* 0.761). Additionally, there was a statistically significant interaction between the TDP-43^M337V^ expression and DOX reduction time on hind paw stride length (distance between successive placements of the same paw), *n* ≥ 6/group, (F (_4, 100_) = 2.940, *p =* 0.024; Fig. 1g). However, there was no significant main effect of time, (F (_4, 100_) = 0.938, *p =* 0.445) while there was an effect of genotype (F (_1, 25_) = 5.616, *p =* 0.026), suggesting that ChAT-tTA/TRE-TDP-43^M337V^ animals generally took smaller strides with DOX reduction over time compared to TDP-43 controls. Interestingly, there was a statistically significant interaction between the TDP-43^M337V^ expression and DOX reduction time on body speed (*n* ≥ 6/group, F (_3.2, 73_) = 2.873, *p =* 0.039; Fig. 1h), with no main effect of time (F (_3.2, 73_) = 1.684, *p =* 0.175) or genotype (F (_1, 23_) = 2.712, *p =* 0.113), implicating that ChAT-tTA/TRE-TDP-43^M337V^ animals reduced their speed with DOX reduction over time, compared to TDP-43 controls (see Additional file 3: Figure S3A-B for other CatWalk results).

Startle box testing revealed no main interactions between genotype or tau and DOX reduction time on baseline startle or PPI (*p >* 0.05; Additional file 4: Figure S4A-D). While ANOVA detected a main effect of genotype on PPI (*p =* 0.047; Additional file 4: Figure S4C), this effect cannot be deduced to DOX reduction over time as ChAT-tTA/TRE-TDP-43^M337V^ rats displayed generally lower PPI, even at baseline, with no main effect of time (*p >* 0.05; see Additional file 4: Figure S4A-D for ASR results, see also Additional file 8).

The expression of any tau type had no effect on distance travelled or thigmotaxis (Additional file 5: Figure S5), though there was a significant interaction between the TDP-43^M337V^ expression on DOX reduction time on thigmotaxis where ChAT-tTA/TRE-TDP-43^M337V^ rats generally spent more time in the center of the open field than the TDP-43 controls (*p* = 0.022; Additional file 5: Figure S5C.

There were no significant main effects found for social recognition or sociability testing (*p* > 0.05; see Additional file 6: Figure S6A-D for social test results).

### Neuroimaging

No differences between groups were observed in either the corpus callosum or hippocampus by neuroimaging as measured by ODI, NDI, or IsoVF (Additional file 7: Figure S7).

## Discussion

By using a rat model in which the expression of pathological TDP-43 can be modulated, we have determined that animals expressing a pathological tau species (tau^T175D^) exhibit increased tau pathology in the hippocampus, even when the expression of TDP-43 is in distant cells. Behaviour and motor tests showed that the expression of TDP-43 did lead to a predictable and detectable motor dysfunction as expected. Our studies have demonstrated that the co-expression of two of the most common hallmark neuropathological proteins associated with neurodegeneration can behave in a synergistic manner even when acting at a distance. This exacerbation was accompanied by markers of neuroinflammation and apoptosis, suggesting that the dual effect of two pathological proteins produces an increase in systems-level toxic changes in the central nervous system.

The exacerbation of tau pathology by a toxic variant of TDP-43 adds another level of complexity to the role of tau protein as either a principal or secondary toxic protein in the neurodegenerative process. The induction of tau protein pathology by cell stressors has been suggested to be a mechanism of tau pathology in traumatic brain injury and Alzheimer’s disease among others [1]. After induction, tau protein has the capacity to feed forward, generating further tau toxicity. Our experiments suggest that other toxic proteins associated with neurodegeneration (in this case TDP-43) can exacerbate tau pathology. While our studies do not allow for a differentiation of a primary tauopathy from a secondary tauopathy as a response to cellular injury, it would be reasonable to expect that the effect will be the same with a further induction of the tauopathy. This is of particular relevance in that tau pathology is frequently observed alongside other pathogenic protein deposition. While this current series of experiments has focused on the development of an in vivo model of ALSci, it is of note that we have also shown pathogenic phospho-Thr^175^ tau protein deposition in a series of neurodegenerative diseases [24]. This concept may be extended to Alzheimer’s disease, Lewy body dementia, and any neurodegenerative disease where tau protein may be comorbid with another proteinopathy [3].

It has been recently reported that tau protein and TDP-43 can be observed to co-localize within RNA granules [15]. In this case, tau metabolism may be directly impacted by pathological TDP-43 contributing to further tau pathology. This potential interaction between tau and RNA binding proteins has also been supported by the observation of a more aggressive tau pathology in a clinical case with both *fused in sarcoma* (FUS) and tau pathology [10, 36].

Of particular interest in our study was the observation that the increase in cortical tau^T175D^ pathology was not dependant on the co-expression of pathologic tau and TDP-43 within the same cell populations, suggesting that an intermediary cell population or factor(s) may drive these changes. It is noteworthy therefore that we observed the presence of hippocampal microglial activation in the TDP-43^M337V^ expressing rats. While the concept that microglial activation can drive a more prominent tauopathy has been previously suggested, it is not clear by what mechanism this may occur [38]. Further while a direct effect through cytokine release could be postulated, we have also observed a trend towards an increase in spinal motor neuron pathology in ChAT-tTA/TRE-TDP-43^M337V^ rats expressing rAAV9 constructs in the hippocampus. This occurred in the absence of obvious corticospinal tract degeneration and thus it seems unlikely to be related to enhanced trans-synaptic degeneration. Rather, in both the cortical and spinal motor neuron pathology, synergistic pathology factors acting both locally and at a distance such as exosomes could be postulated. While spinal fluid was not collected in this current study, future studies will do so in order to further clarify the mechanism(s) of pathology synergism that we have observed.

This study was limited by low numbers as animals were extremely challenging to generate and carrying them to the required time point was not easy due to health challenges encountered by aged animals in this line [22]. This limited the power of neuroimaging and behavioural studies to detect differences between groups. Regardless, age is a challenging component of neurodegenerative disease to model and the adult onset expression of two pathological proteins at advanced age was critical to the question asked in this study. Surprisingly, we did not detect any behavioural changes in these rats, even though the anticipated motor phenotype manifested as expected, and pathology was increased with the expression of both proteins. However, this was a comparatively short study after the induction of TDP-43 expression, and it is possible that changes were beginning but needed more time to manifest completely. Both neuroimaging and behavioural analyses may also lack the sensitivity necessary to detect the subtle changes in small brain regions as observed in this study. For neuroimaging, the digital masking of the CA2 region of the hippocampus was not possible due to the small size of the brain structure, so assessment of this region by NODDI was not possible. Irrespective of this inability to detect subtle changes, the observed enhancement effect of the neuropathology in this model warrants further investigation.

The DOX used in this model may have introduced a confounding factor through an unintended neuroprotective effect [29]. This possibility, in the light of the increase in neuropathology, might suggest that our results have underestimated the impact of the synergistic effect of expressing two toxic variants of neurodegeneration-related proteins.

The synergy between two pathological proteins in the same nervous system may be cause to reconsider the framework used to classify neurodegenerative disease for therapeutic intervention. Historically, attempts to connect different clinical phenotypes with a specific type of protein inclusion have been made. This has led to increasingly complex systems of classification which aim to stratify clinical presentation based on the predominant types of inclusion (protein composition and morphology) [26]. Our findings would suggest that this framework is inadequate for understanding of the mechanisms underlying the progressive neuronal death in neurodegenerative disease. Indeed, the presence of comorbid pathologies is increasingly the rule rather than the exception, complicating the neuropathological and clinical correlations. We have also previously suggested that the location of neuronal death rather than specific protein is likely to be responsible for clinical presentation of disease and that any toxic protein that may disturb the same population of neurons will generate the same clinical phenotype [23]. Effective therapeutic intervention may only be possible when accounting for the coalescence of pathologies in each individual case, irrespective of clinical presentation.

Our observations are of specific importance to our understanding of ALS with an accompanying frontotemporal dysfunction. Specifically, the trend towards aninduction of greater spinal motor neuron pathology in the presence of neocortical tau pathology may provide an insight into the more rapid disease course observed in ALS when a frontotemporal dementia phenotype is present [7, 11, 18]. Importantly, these two clinical manifestations are associated with TDP-43 and tau protein pathological deposition in the spinal cord and brain, respectively. Therefore, beyond shifting the understanding of the disease process itself, the finding that the presence of synergistic neuropathology in the disease process may shed light on clinical observations that remain unexplained and have important implications for prognosis in this population. While synergy between proteins has been described previously (particularly for Alzheimer’s disease between amyloid-beta and tau [27] and potentially involving prion-proteins as well [27]), our data support the concept that the net effect of expressing two pathogenic proteins within the nervous system, even if such proteins are expressed in different cell types, is a synergistic increase in pathology. Given that this model may more fully reflect the pathology of neurodegeneration in affected humans, future studies should employ such a model when examining for therapeutic drug efficacy.

## Conclusion

In this series of experiments, we have extended our previous observations to confirm the in vivo pathogenecity of Thr175Asp tau (tau^T175D^;a pseudophosphorylated tau construct mimicking pThr175 tau) and to demonstrate that the co-expression of mutant TDP-43 (TDP-43^M337V^) acts synergistically to significantly increase the neuropathological features of this experimental tauopathy. Critically, this synergism does not require the co-localization of either pathogenic protein within the same cells and indeed, there is evidence that the neocortical pathology induced by the tau^T175D^ augments the spinal motor neuron pathology observed as a consequence of the expression of TDP-43^M337V^. While the mechanism by which this synergism occurs remains the subject of ongoing experiments, these observations have important consequences on our understanding of the impact of dual pathologies in a broad range of neurodegenerative disorders.

## Supplementary information

**Additional file 1:** **Figure S1.** Schematic depicting the rat model used in the TDP-43^M337V^ experiments. A) Cartoon representation of background (Sprague-Dawley) line used to generate ChAT-tTA (Choline acetyltransferase promoter paired with tetracycline transactivator promoter) and TRE-TDP-43^M337V^ (tetracycline response element paired with mutant human TDP-43^M337V^) non-expressor lines. Crossing these two lines generates the ChAT-tTA/TRE-TDP-43^M337V^ animals used in experiments. B) Cartoon representation of TDP-43^M337V^ suppression by tTA in the presence of doxycycline (DOX) and expression in the absence of doxycyline.

**Additional file 2:** **Figure S2.** Astrogliosis is not elevated in the hippocampus of ChAT-tTA/TRE-TDP-43^M337V^ rats expressing tau^T175D^. A) GFAP staining in ChAT-tTA rat hippocampus. B) GFAP staining in tau^T175D^ expressing hippocampus on the TDP-43 expressing genetic background. C) Quantification of GFAP positive field of view shows no statistical difference in coverage (proxy for astrocytic activation) in ChAT-tTA/TRE-TDP-43^M337V^ rats expressing any GFP construct (GFP = green fluorescent protein, tau^WT^ = GFP-tagged tau^WT^; tau^T175D^ = GFP-tagged tau^T175D^) in the hippocampus. All quantification represents GFP-tau expressing group on ChAT-tTA/TRE-TDP-43^M337V^ rats’ transgenic background. Images taken using 20x objective. Scale bar = 50 μm.

**Additional file 3:** **Figure S3.** CatWalk hind paw stride length and body speed were disrupted in ChAT-tTA/TRE-TDP-43^M337V^ rats upon doxycycline (DOX) reduction over time. A) The interaction between the TDP-43^M337V^ and DOX reduction time on hind paw swing; the duration of time the paw was not in contact with the glass plate was not significant (*n* ≥ 6/group, *p =* 0.178). B) There was no statistically significant interaction between the TDP-43^M337V^ and DOX reduction time on hind paw swing speed (speed of the paw while taking a step), however we were approaching significance (*n* ≥ 6/group, *p =* 0.059).

**Additional file 4:** **Figure S4.** Startle and prepulse inhibition were unaffected by 50% doxycycline (DOX) reduction. A) Startle box testing revealed no significant interaction between TDP-43^M337V^ expression and DOX reduction over time on baseline startle response, (*n* ≥ 6/group, *p =* 0.772). There was no statistically significant difference in mean baseline startle between ChAT-tTA/TRE-TDP-43^M337V^ and TDP-43 controls (*p =* 0.364). B) Within ChAT-tTA/TRE-TDP-43^M337V^ rats there was no significant interaction between the tau AAV9 vector injection groups and time on baseline startle response (*p =* 0.097). C) No interaction between TDP-43^M337V^ and time on PPI (*p =* 0.868). There was a significant difference in mean PPI between ChAT-tTA/TRE-TDP-43^M337V^ and TDP-43 controls (*p =* 0.047), where ChAT-tTA/TRE-TDP-43^M337V^ animals generally had lower levels of PPI than the control group, regardless of the time point. *p = 0.047. D) Within ChAT-tTA/TRE-TDP-43^M337V^ rats there was no significant main interaction of tau AAV9 vector injection groups and time on PPI (*p =* 0.607). The main effect of time showed no statistically significant difference on mean PPI (*p =* 0.065). There was no statistically significant difference in mean PPI between injection groups (*p =* 0.314). GFP = green fluorescent protein, tau^WT^ = GFP-tagged tau^WT^, tau^T175D^ = GFP-tagged human tau^T175D^.

**Additional file 5:** **Figure S5.** Open field testing. A) No significant interaction was identified by mixed ANOVA between TDP-43^M337V^ and doxycycline (DOX) reduction over time on cumulative distance traveled, (*n* ≥ 5/group, *p =* 0.161). Similarly, there were no main effects of time (*p =* 0.421) or genotype (*p =* 0.502) on cumulative distance traveled. B) Within ChAT-tTA/TRE-TDP-43^M337V^ rats, there were no significant main effects of time (*p =* 0.824), tau injection groups (*p =* 0.085), or the interaction between the two on cumulative distance traveled (*n* ≥ 3/group, *p =* 0.300). C) Mixed ANOVA revealed a significant interaction between TDP-43^M337V^ and DOX reduction over time on thigmotaxis, (*n* ≥ 4/group, *p =* 0.022). The main effect of time also showed a statistically significant difference in mean thigmotaxis at the different time points, (*p =* 0.039), however post hoc t-tests with Bonferroni correction revealed no significant differences between any of the time points (*p ≥* 0.05). There was also a significant difference in mean thigmotaxis between ChAT-tTA/TRE-TDP-43^M337V^ and TDP-43 controls (*p =* 0.039) regardless of the time, suggesting that control animal generally spent more time in the perimeter as opposed to the centre of the open-field (* *p ≤* 0.05). D) Within ChAT-tTA/TRE-TDP-43^M337V^ rats, there was no significant interaction between tau and time on thigmotaxis, (*n* ≥ 3/group, *p =* 0.240). Main effect of time showed a statistically significant difference in mean thigmotaxis at the different time points (*p =* 0.001) and post hoc t-tests with Bonferroni correction revealed a significant difference between baseline (before DOX reduction) and 2 weeks following DOX reduction (*p =* 0.004). There was no statistically significant difference in mean thigmotaxis between injection groups (*p =* 0.383). GFP = green fluorescent protein, WT-tau = GFP-tagged wild-type human tau, Thr^175^Asp-tau = GFP-tagged Thr^175^Asp human tau. *p ≤ 0.05.

**Additional file 6:** **Figure S6.** Sociability and social novelty. A) Mixed ANOVA found no statistically significant interaction between TDP-43^M337V^ and time on sociability difference score, (*n* ≥ 3/group, *p =* 0.413). There was also no main effect of time (*p =* 0.426) or genotype (*p =* 0.473) on sociability difference score. B) No statistically significant effect of time (*p =* 0.757), genotype (*p =* 0.495), or the interaction between the two on sociability difference score (*n* ≥ 2/group, *p =* 0.102). C) No statistically significant interaction between TDP-43^M337V^ and time on social preference difference score (*n* ≥ 2/group, *p =* 0.101). The main effect of time showed no statistically significant difference in social preference difference score at the different time points (*p =* 0.232). There was also no statistically significant difference in mean social preference difference score between ChAT-tTA/TRE-TDP-43^M337V^ and TDP-43 controls (*p =* 0.163). D) No statistically significant interaction between tau AAV9 injection groups and DOX reduction time on social preference difference score (*n* ≥ 2/group, *p =* 0.535). The main effect of time showed no statistically significant difference in mean social preference difference score at the different time points (*p =* 0.296). There was also no statistically significant difference in mean social preference difference score between injection groups (*p =* 0.295). GFP = green fluorescent protein, tau^WT^ = GFP-tagged human tau^WT^, tau^T175D^ = GFP-tagged human tau^T175D^.

**Additional file 7:** **Figure S7.** Neurite Orientation Dispersion and Density Imaging (NODDI) diffusion weighted magnetic resonance imaging (dMRI) in transgenic rats. A) Representative images showing Orientation Dispersion Index (ODI) Neurite Density Index (NDI), and the CSF volume fraction (IsoVF). B) No differences were observed between rAAV9-GFP-tau construct groups in ODI, NDI, or IsoVF in the corpus callosum. C) No differences were observed between GFP-tau construct groups in ODI, NDI, or IsoVF in the hippocampus. Data represent mean of four animals per group. GFP = green fluorescent protein, tau^WT^ = GFP-tagged human tau^WT^, tau^T175D^ = GFP-tagged human tau^T175D^.

**Additional file 8.** Behavioural results.

## Data Availability

The datasets generated for the behaviour studies can be requested from the corresponding author.

## Abbreviations

ALS: Amyotrophic lateral sclerosis
ALSci: Amyotrophic lateral sclerosis with cognitive impairment
ANOVA: Analysis of variance
ASR: Acoustic startle response
AUP: Animal use protocol
BSA: Bovine serum albumin
ChAT: Choline acetyltransferase
DAB: 3,3′-diaminobenzidine
dB: Decibel
dMRI: Diffusion magnetic resonance imaging
DOX: Doxycycline
EPI: Echo-planar imaging
FTSD: Frontotemporal spectrum disorder
GFAP: Glial fibrillary acidic protein
GFP: Green fluorescent protein
H&E: Hematoxylin and eosin
IHC: Immunohistochemistry
ISI: Inter stimulus interval
isoVF: Volume fraction of Gaussian isotropic diffusion
MRI: Magnetic resonance imaging
NDI: Neurite density index
NODDI: Neurite orientation dispersion and density imaging
ODI: Orientation dispersion index
OFT: Open field test
PBS: Phosphate buffered saline
PPI: Prepulse inhibition
rAAV9: Recombinant adeno-associated virus subtype 9
SD: Sprague-Dawley
TDP-43: TAR DNA binding protein of 43 kDa
TRE: Tetracycline response element
tTA: Tetracycline-controlled transactivator
WT: Wild type

## References

[1] Alavi Naini SM, Soussi-Yanicostas N (2015). Tau hyperphosphorylation and oxidative stress, a critical vicious circle in neurodegenerative tauopathies?. Oxidative Med Cell Longev.

[2] Armstrong DM, Saper CB, Levey AI, Wainer BH, Terry RD (1983). Distribution of cholinergic neurons in rat brain: demonstrated by the immunocytochemical localization of choline acetyltransferase. J Comp Neurol.

[3] Chornenkyy Yevgen, Fardo David W., Nelson Peter T. (2019). Tau and TDP-43 proteinopathies: kindred pathologic cascades and genetic pleiotropy. Laboratory Investigation.

[4] Clinton LK, Blurton-Jones M, Myczek K, Trojanowski JQ, LaFerla FM (2010). Synergistic interactions between Aβ, tau, and α-synuclein: acceleration of neuropathology and cognitive decline. J Neurosci.

[5] Consonni S, Leone S, Becchetti A, Amadeo A (2009). Developmental and neurochemical features of cholinergic neurons in the murine cerebral cortex. BMC Neurosci.

[6] Devito LM, Konigsberg R, Lyyken C, Sauvage M, Scott W, Eichenbaum H (2009). Vasopressin 1b receptor knockout impairs memory for temporal order. J Neurosci.

[7] Elamin M, Bede P, Byrne S, Jordan N, Gallagher L, Wynne B (2013). Cognitive changes predict functional decline in ALS: a population-based longitudinal study. Neurology..

[8] Factor-Litvak P, Al-Chalabi A, Ascherio A, Bradley W, Chío A, Garruto R (2013). Current pathways for epidemiological research in amyotrophic lateral sclerosis. Amyotroph Lateral Scler Front Degener.

[9] Gohar M, Yang W, Strong W, Volkening K, Leystra-Lantz C, Strong MJ (2009). Tau phosphorylation at threonine-175 leads to fibril formation and enhanced cell death: implications for amyotrophic lateral sclerosis with cognitive impairment. J Neurochem.

[10] Gomes LA, Hipp SA, Upadhaya AR, Balakrishnan K, Ospitalieri S, Koper MJ et al (2019) Aβ-induced acceleration of Alzheimer-related τ-pathology spreading and its association with prion protein. Acta Neuropathol. 10.1007/S00401-019-02053-5 10.1007/s00401-019-02053-5 31414210

[11] Gordon PH, Goetz RR, Rabkin JG, Dalton K, McElhiney M, Hays AP (2010). A prospective cohort study of neuropsychological test performance in ALS. Amyotroph Lateral Scler.

[12] Higashi S, Iseki E, Yamamoto R, Minegishi M, Hino H, Fujisawa K (2007). Concurrence of TDP-43, tau and α-synuclein pathology in brains of Alzheimer’s disease and dementia with Lewy bodies. Brain Res.

[13] Hitti FL, Siegelbaum SA (2014). The hippocampal CA2 region is essential for social memory. Nature..

[14] Huang C, Tong J, Bi F, Zhou H, Xia XG (2012). Mutant TDP-43 in motor neurons promotes the onset and progression of ALS in rats. J Clin Invest.

[15] Jiang L, Ash PEA, Maziuk BF, Ballance HI, Boudeau S, Abdullatif AA (2019). TIA1 regulates the generation and response to toxic tau oligomers. Acta Neuropathol.

[16] Lauer AM, Behrens D, Klump G (2017). Acoustic startle modification as a tool for evaluating auditory function of the mouse: progress, pitfalls, and potential. Neurosci Biobehav Rev.

[17] Ling SC, Polymenidou M, Cleveland DW (2013). Converging mechanisms in als and FTD: disrupted RNA and protein homeostasis. Neuron..

[18] Lomen-Hoerth C (2011). Clinical phenomenology and neuroimaging correlates in ALS-FTD. J Mol Neurosci.

[19] Lu Y, Sun XD, Hou FQ, Bi LL, Yin DM, Liu F (2014). Maintenance of GABAergic activity by neuregulin 1-ErbB4 in amygdala for fear memory. Neuron..

[20] McCunn P, Gilbert KM, Zeman P, Li AX, Strong MJ, Khan AR (2019). Reproducibility of neurite orientation dispersion and density imaging (NODDI) in rats at 9.4 tesla. PLoS One.

[21] Moszczynski AJ, Gohar M, Volkening K, Leystra-Lantz C, Strong W, Strong MJ (2015). Thr175-phosphorylated tau induces pathologic fibril formation via GSK3β-mediated phosphorylation of Thr231 invitro. Neurobiol Aging.

[22] Moszczynski AJ, Gopaul J, McCunn P, Volkening K, Harvey M, Bartha R (2018). Somatic gene transfer using a recombinant adenoviral vector (rAAV9) encoding pseudophosphorylated human Thr175 tau in adult rat hippocampus induces tau pathology. J Neuropathol Exp Neurol.

[23] Moszczynski AJ, Strong MJ, Cechetto D, Weishaupt N (2016). Cortical manifestations in amyotrophic lateral sclerosis. Cereb cortex neurodegener neuropsychiatr disord.

[24] Moszczynski AJ, Yang W, Hammond R, Ang LC, Strong MJ (2017). Threonine175, a novel pathological phosphorylation site on tau protein linked to multiple tauopathies. J Intensive Care.

[25] Mustroph ML, King MA, Klein RL, Ramirez JJ (2012). Adult-onset focal expression of mutated human tau in the hippocampus impairs spatial working memory of rats. Behav Brain Res.

[26] Neumann M, Mackenzie IRA (2019). Review: neuropathology of non-tau frontotemporal lobar degeneration. Neuropathol Appl Neurobiol.

[27] Pascoal TA, Mathotaarachchi S, Mohades S, Benedet AL, Chung CO, Shin M (2017). Amyloid-β and hyperphosphorylated tau synergy drives metabolic decline in preclinical Alzheimer’s disease. Mol Psychiatry.

[28] Rydkjaer J, Jepsen JRM, Pagsberg AK, Fagerlund B, Glenthoej BY, Oranje B (2019) Do young adolescents with first-episode psychosis or ADHD show sensorimotor gating deficits? Psychol Med. 10.1017/S0033291719000412 10.1017/S0033291719000412 30873927

[29] Santa-Cecília FV, Leite CA, Del-Bel E, Raisman-Vozari R (2019). The neuroprotective effect of doxycycline on neurodegenerative diseases. Neurotox Res.

[30] Soma K, Fu YJ, Wakabayashi K, Onodera O, Kakita A, Takahashi H (2012). Co-occurrence of argyrophilic grain disease in sporadic amyotrophic lateral sclerosis. Neuropathol Appl Neurobiol.

[31] Strong MJ, Abrahams S, Goldstein LH, Woolley S, Mclaughlin P, Snowden J (2017). Amyotrophic lateral sclerosis - frontotemporal spectrum disorder (ALS-FTSD): revised diagnostic criteria. Amyotroph Lateral Scler Front Degener.

[32] Strong MJ, Yang W, Strong WL, Leystra-Lantz C, Jaffe H, Pant HC (2006). Tau protein hyperphosphorylation in sporadic ALS with cognitive impairment. Neurology..

[33] Takahashi H, Kamio Y (2018). Acoustic startle response and its modulation in schizophrenia and autism spectrum disorder in Asian subjects. Schizophr Res.

[34] Valsamis B, Schmid S (2011) Habituation and prepulse inhibition of acoustic startle in rodents. J Vis Exp JoVE. 10.3791/3446 10.3791/3446 PMC3217252 21912367

[35] Vergouts M, Marinangeli C, Ingelbrecht C, Genard G, Schakman O, Sternotte A (2015). Early ALS-type gait abnormalities in AMP-dependent protein kinase-deficient mice suggest a role for this metabolic sensor in early stages of the disease. Metab Brain Dis.

[36] Wharton S. B., Verber N. S., Wagner B. E., Highley J. R., Fillingham D. J., Waller R., Strand K., Ince P. G., Shaw P. J. (2019). Combined fused in sarcoma‐positive (FUS+) basophilic inclusion body disease and atypical tauopathy presenting with an amyotrophic lateral sclerosis/motor neurone disease (ALS/MND)‐plus phenotype. Neuropathology and Applied Neurobiology.

[37] Yang W, Strong MJ (2012). Widespread neuronal and glial hyperphosphorylated tau deposition in ALS with cognitive impairment. Amyotroph Lateral Scler.

[38] Yoshiyama Y, Higuchi M, Zhang B, Huang SM, Iwata N, Saido TCC (2007). Synapse loss and microglial activation precede tangles in a P301S tauopathy mouse model. Neuron..

[39] Zhang H, Hubbard P, Parker G, Alexander D (2011). Axon diameter mapping in the presence of orientation dispersion with diffusion MRI. Neuroimage..

[40] Zhang H, Schneider T, Wheeler-Kingshott CA, Alexander DC (2012). NODDI: practical in vivo neurite orientation dispersion and density imaging of the human brain. Neuroimage..

[41] Zhou H, Huang C, Chen H, Wang D, Landel CP, Xia PY (2010). Transgenic rat model of neurodegeneration caused by mutation in the TDP gene. PLoS Genet.

[42] Zhou H, Huang C, Yang M, Landel CP, Xia PY, Liu YJ (2009). Developing tTA transgenic rats for inducible and reversible gene expression. Int J Biol Sci.

