# Supplementary figures and images for: Synergistic toxicity in an in vivo model of neurodegeneration through the co-expression of human TDP-43M337V and tauT175D protein
Source: Acta Neuropathol Commun. 2019 Nov 8;7:170. doi: 10.1186/s40478-019-0816-1 (PMC6839082; doi:10.1186/s40478-019-0816-1)

**A**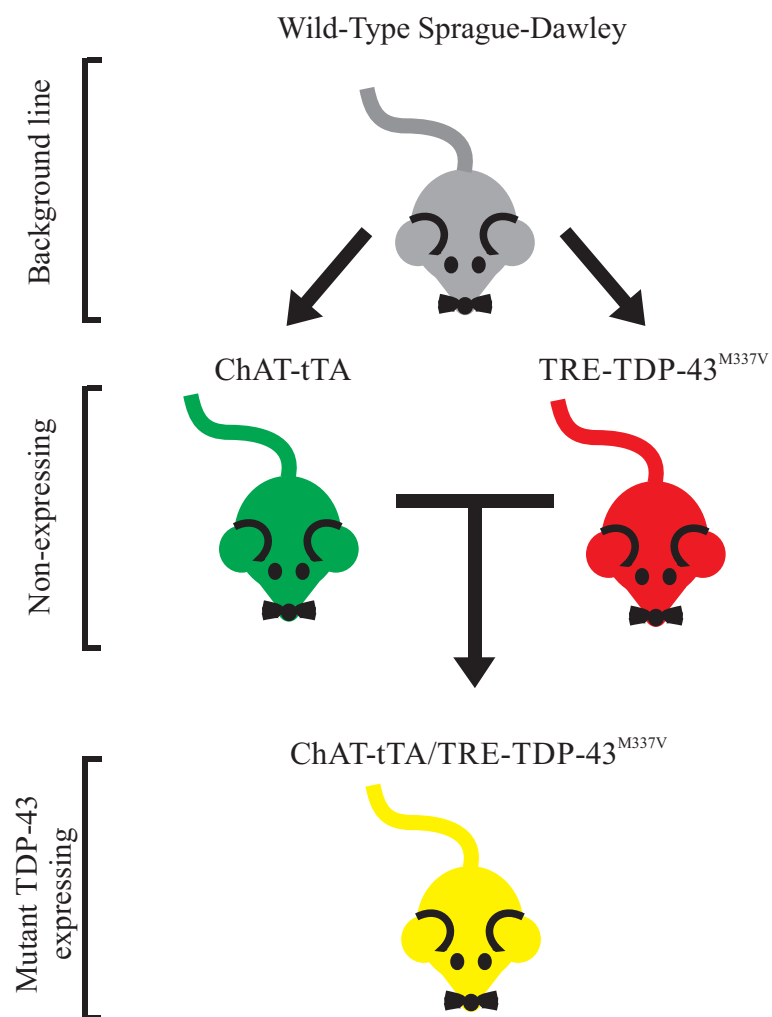**B**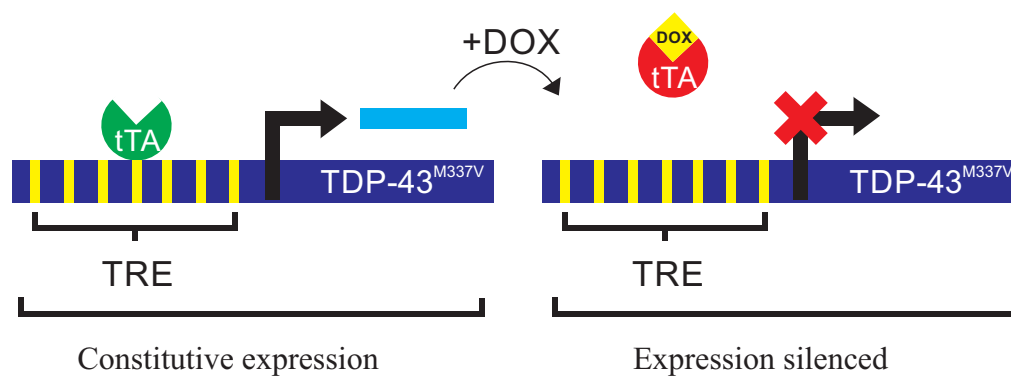

Supplement: Supplementary file 1 — Additional file 1: Figure S1. Schematic depicting the rat model used in the TDP-43M337V experiments. A) Cartoon representation of background (Sprague-Dawley) line used to generate ChAT-tTA (Choline acetyltransferase promoter paired with tetracycline transactivator promoter) and TRE-TDP-43M337V (tetracycline response element paired with mutant human TDP-43M337V) non-expressor lines. Crossing these two lines generates the ChAT-tTA/TRE-TDP-43M337V animals used in experiments. B) Cartoon representation of TDP-43M337V suppression by tTA in the presence of doxycycline (DOX) and expression in the absence of doxycyline. [file 40478_2019_816_MOESM1_ESM.pdf]

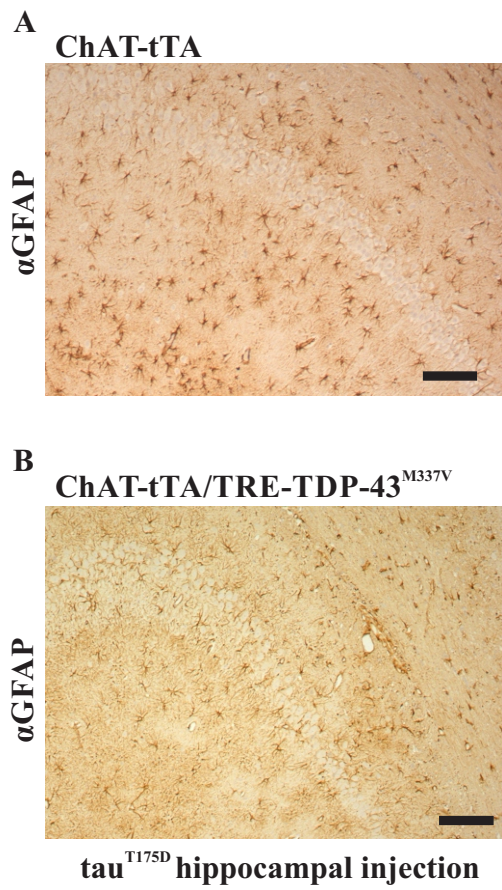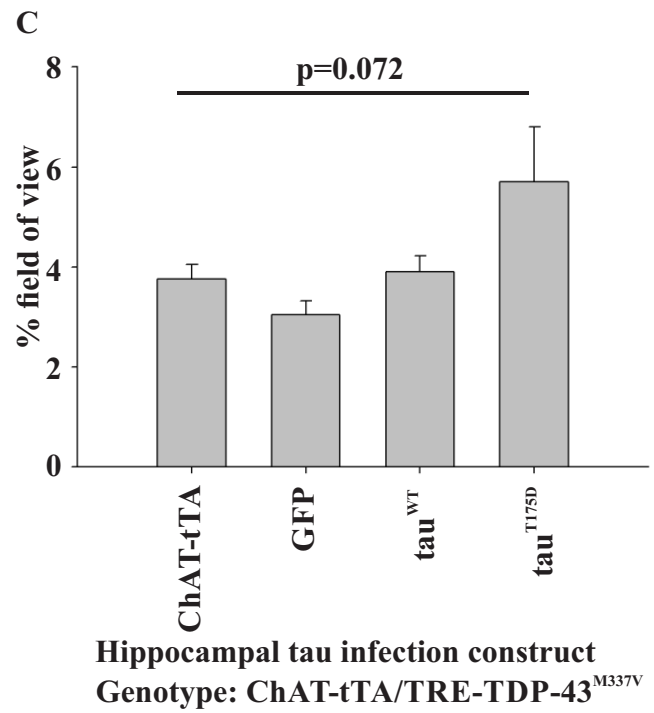

Supplement: Supplementary file 2 — Additional file 2: Figure S2. Astrogliosis is not elevated in the hippocampus of ChAT-tTA/TRE-TDP-43M337V rats expressing tauT175D. A) GFAP staining in ChAT-tTA rat hippocampus. B) GFAP staining in tauT175D expressing hippocampus on the TDP-43 expressing genetic background. C) Quantification of GFAP positive field of view shows no statistical difference in coverage (proxy for astrocytic activation) in ChAT-tTA/TRE-TDP-43M337V rats expressing any GFP construct (GFP = green fluorescent protein, tauWT = GFP-tagged tauWT; tauT175D = GFP-tagged tauT175D) in the hippocampus. All quantification represents GFP-tau expressing group on ChAT-tTA/TRE-TDP-43M337V rats’ transgenic background. Images taken using 20x objective. Scale bar = 50 μm. [file 40478_2019_816_MOESM2_ESM.pdf]

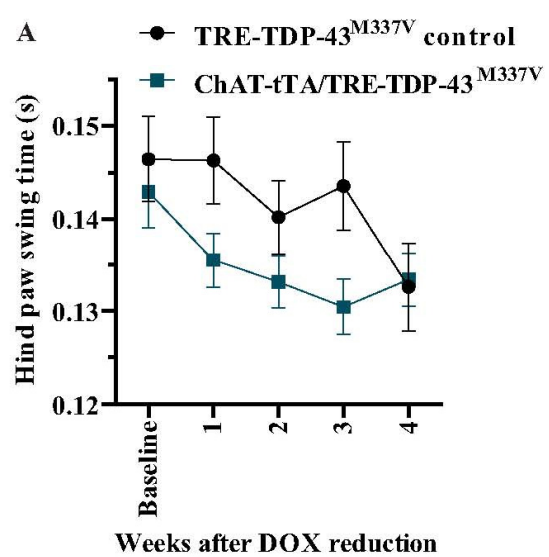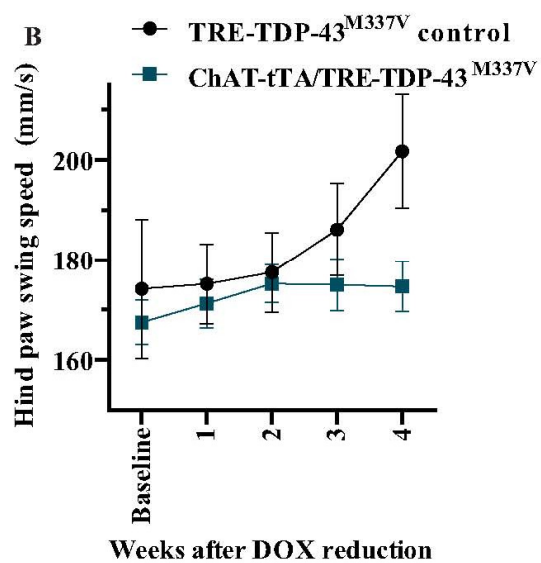

Supplement: Supplementary file 3 — Additional file 3: Figure S3. CatWalk hind paw stride length and body speed were disrupted in ChAT-tTA/TRE-TDP-43M337V rats upon doxycycline (DOX) reduction over time. A) The interaction between the TDP-43M337V and DOX reduction time on hind paw swing; the duration of time the paw was not in contact with the glass plate was not significant (n ≥ 6/group, p = 0.178). B) There was no statistically significant interaction between the TDP-43M337V and DOX reduction time on hind paw swing speed (speed of the paw while taking a step), however we were approaching significance (n ≥ 6/group, p = 0.059). [file 40478_2019_816_MOESM3_ESM.pdf]

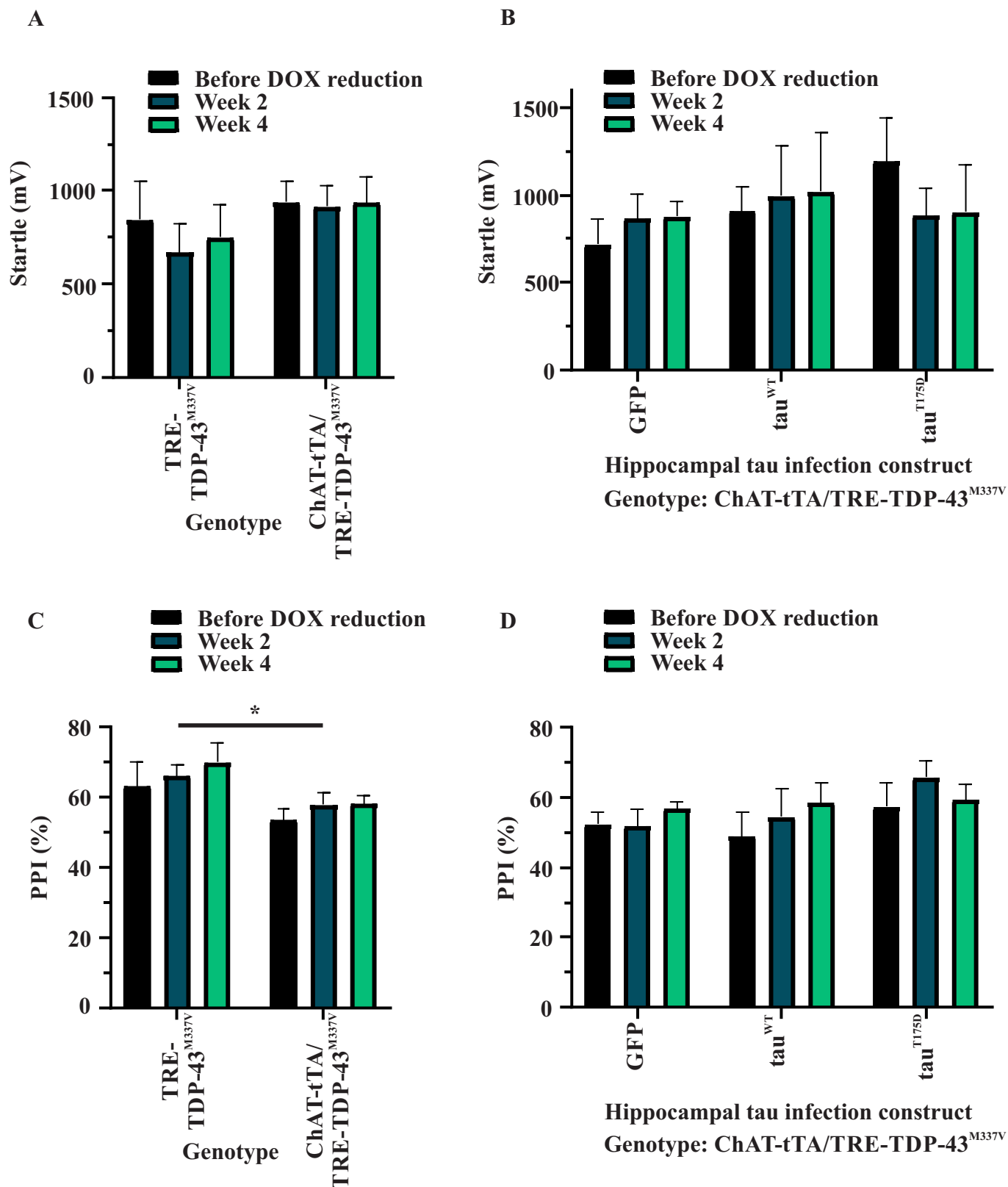

Supplement: Supplementary file 4 — Additional file 4: Figure S4. Startle and prepulse inhibition were unaffected by 50% doxycycline (DOX) reduction. A) Startle box testing revealed no significant interaction between TDP-43M337V expression and DOX reduction over time on baseline startle response, (n ≥ 6/group, p = 0.772). There was no statistically significant difference in mean baseline startle between ChAT-tTA/TRE-TDP-43M337V and TDP-43 controls (p = 0.364). B) Within ChAT-tTA/TRE-TDP-43M337V rats there was no significant interaction between the tau AAV9 vector injection groups and time on baseline startle response (p = 0.097). C) No interaction between TDP-43M337V and time on PPI (p = 0.868). There was a significant difference in mean PPI between ChAT-tTA/TRE-TDP-43M337V and TDP-43 controls (p = 0.047), where ChAT-tTA/TRE-TDP-43M337V animals generally had lower levels of PPI than the control group, regardless of the time point. *p = 0.047. D) Within ChAT-tTA/TRE-TDP-43M337V rats there was no significant main interaction of tau AAV9 vector injection groups and time on PPI (p = 0.607). The main effect of time showed no statistically significant difference on mean PPI (p = 0.065). There was no statistically significant difference in mean PPI between injection groups (p = 0.314). GFP = green fluorescent protein, tauWT = GFP-tagged tauWT, tauT175D = GFP-tagged human tauT175D. [file 40478_2019_816_MOESM4_ESM.pdf]

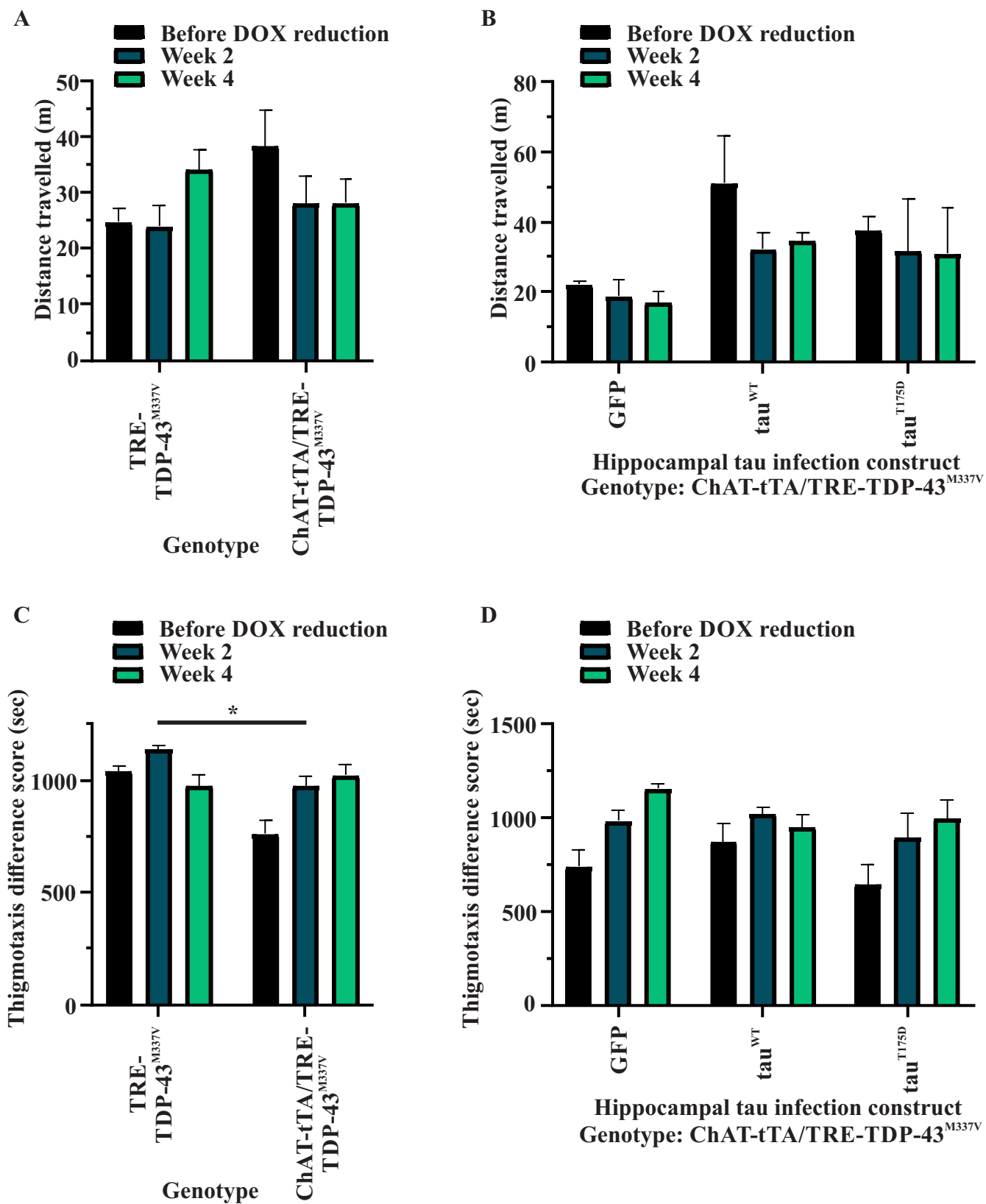

Supplement: Supplementary file 5 — Additional file 5: Figure S5. Open field testing. A) No significant interaction was identified by mixed ANOVA between TDP-43M337V and doxycycline (DOX) reduction over time on cumulative distance traveled, (n ≥ 5/group, p = 0.161). Similarly, there were no main effects of time (p = 0.421) or genotype (p = 0.502) on cumulative distance traveled. B) Within ChAT-tTA/TRE-TDP-43M337V rats, there were no significant main effects of time (p = 0.824), tau injection groups (p = 0.085), or the interaction between the two on cumulative distance traveled (n ≥ 3/group, p = 0.300). C) Mixed ANOVA revealed a significant interaction between TDP-43M337V and DOX reduction over time on thigmotaxis, (n ≥ 4/group, p = 0.022). The main effect of time also showed a statistically significant difference in mean thigmotaxis at the different time points, (p = 0.039), however post hoc t-tests with Bonferroni correction revealed no significant differences between any of the time points (p ≥ 0.05). There was also a significant difference in mean thigmotaxis between ChAT-tTA/TRE-TDP-43M337V and TDP-43 controls (p = 0.039) regardless of the time, suggesting that control animal generally spent more time in the perimeter as opposed to the centre of the open-field (* p ≤ 0.05). D) Within ChAT-tTA/TRE-TDP-43M337V rats, there was no significant interaction between tau and time on thigmotaxis, (n ≥ 3/group, p = 0.240). Main effect of time showed a statistically significant difference in mean thigmotaxis at the different time points (p = 0.001) and post hoc t-tests with Bonferroni correction revealed a significant difference between baseline (before DOX reduction) and 2 weeks following DOX reduction (p = 0.004). There was no statistically significant difference in mean thigmotaxis between injection groups (p = 0.383). GFP = green fluorescent protein, WT-tau = GFP-tagged wild-type human tau, Thr175Asp-tau = GFP-tagged Thr175Asp human tau. *p ≤ 0.05. [file 40478_2019_816_MOESM5_ESM.pdf]

A

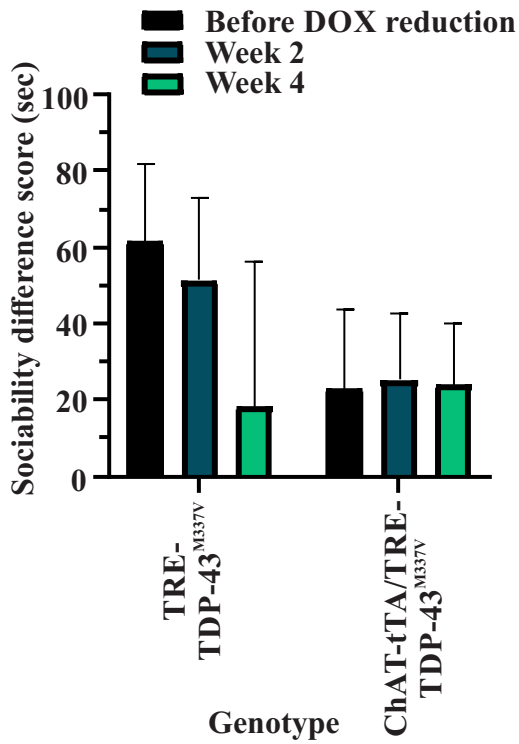

B

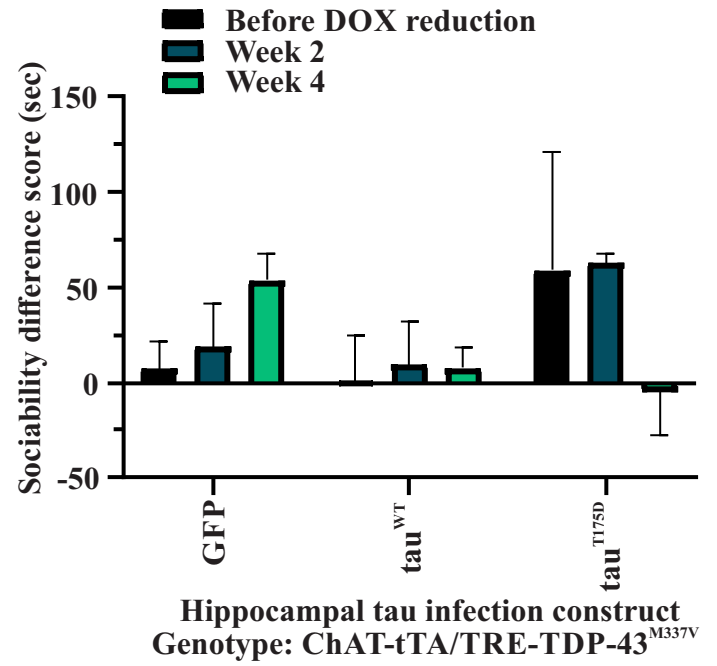

C

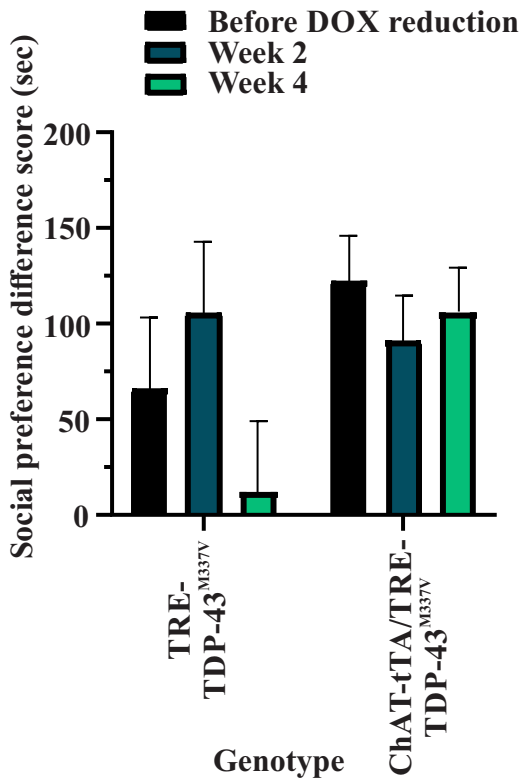

D

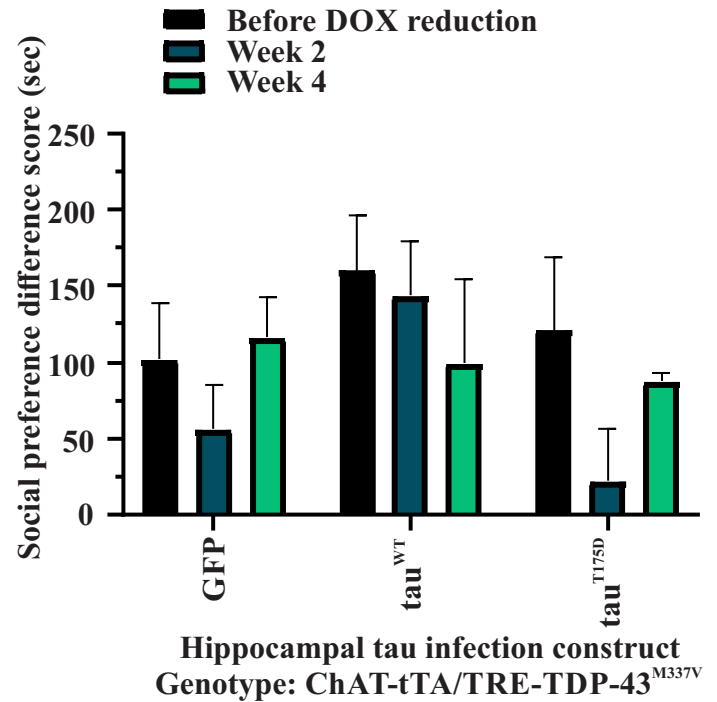

Supplement: Supplementary file 6 — Additional file 6: Figure S6. Sociability and social novelty. A) Mixed ANOVA found no statistically significant interaction between TDP-43M337V and time on sociability difference score, (n ≥ 3/group, p = 0.413). There was also no main effect of time (p = 0.426) or genotype (p = 0.473) on sociability difference score. B) No statistically significant effect of time (p = 0.757), genotype (p = 0.495), or the interaction between the two on sociability difference score (n ≥ 2/group, p = 0.102). C) No statistically significant interaction between TDP-43M337V and time on social preference difference score (n ≥ 2/group, p = 0.101). The main effect of time showed no statistically significant difference in social preference difference score at the different time points (p = 0.232). There was also no statistically significant difference in mean social preference difference score between ChAT-tTA/TRE-TDP-43M337V and TDP-43 controls (p = 0.163). D) No statistically significant interaction between tau AAV9 injection groups and DOX reduction time on social preference difference score (n ≥ 2/group, p = 0.535). The main effect of time showed no statistically significant difference in mean social preference difference score at the different time points (p = 0.296). There was also no statistically significant difference in mean social preference difference score between injection groups (p = 0.295). GFP = green fluorescent protein, tauWT = GFP-tagged human tauWT, tauT175D = GFP-tagged human tauT175D. [file 40478_2019_816_MOESM6_ESM.pdf]

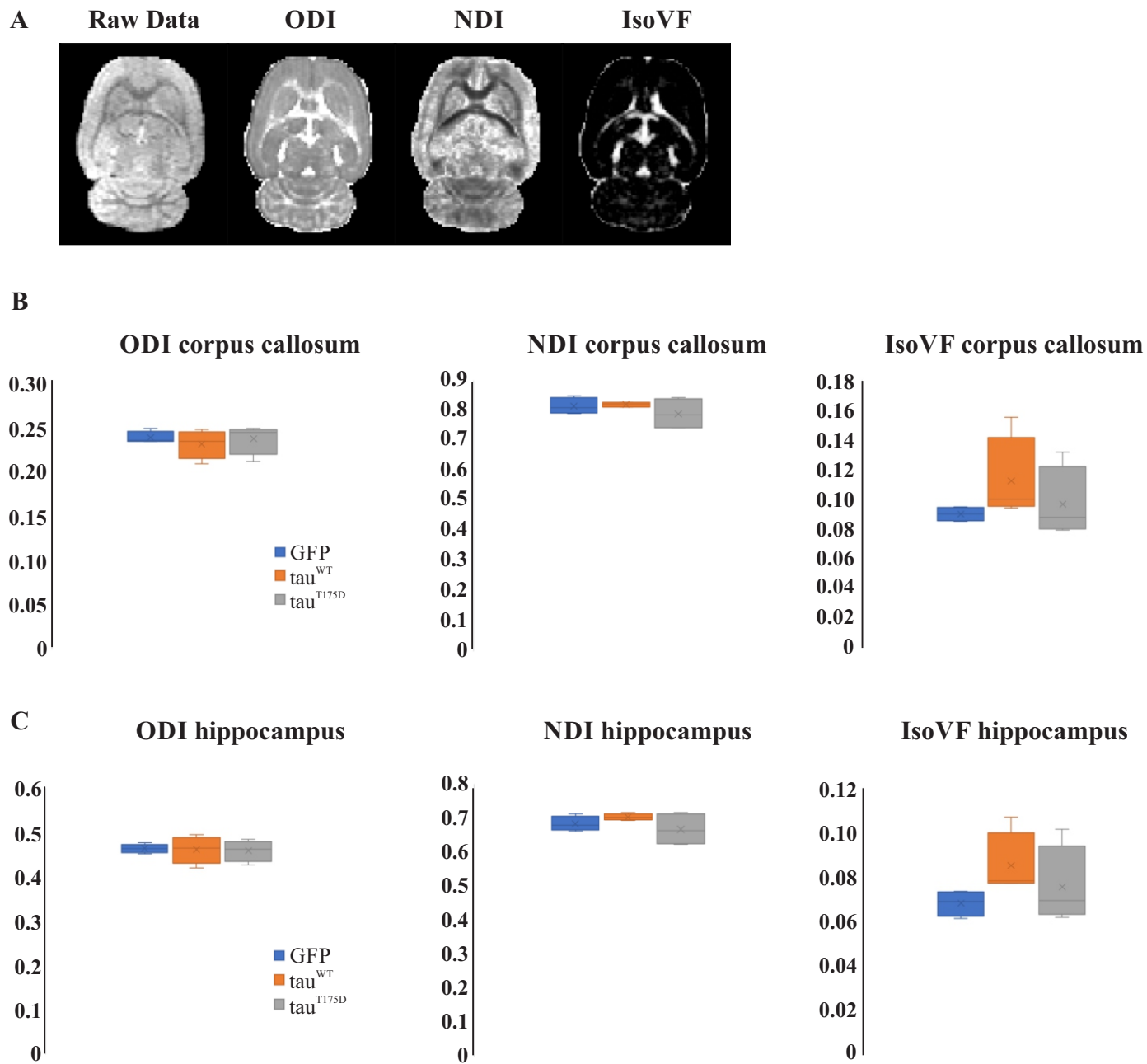

Moszczynski et al, Figure S7

Supplement: Supplementary file 7 — Additional file 7: Figure S7. Neurite Orientation Dispersion and Density Imaging (NODDI) diffusion weighted magnetic resonance imaging (dMRI) in transgenic rats. A) Representative images showing Orientation Dispersion Index (ODI) Neurite Density Index (NDI), and the CSF volume fraction (IsoVF). B) No differences were observed between rAAV9-GFP-tau construct groups in ODI, NDI, or IsoVF in the corpus callosum. C) No differences were observed between GFP-tau construct groups in ODI, NDI, or IsoVF in the hippocampus. Data represent mean of four animals per group. GFP = green fluorescent protein, tauWT = GFP-tagged human tauWT, tauT175D = GFP-tagged human tauT175D. [file 40478_2019_816_MOESM7_ESM.pdf]
