# Supplementary material for: Synergistic toxicity in an in vivo model of neurodegeneration through the co-expression of human TDP-43M337V and tauT175D protein
Source: Acta Neuropathol Commun. 2019 Nov 8;7:170. doi: 10.1186/s40478-019-0816-1 (PMC6839082; doi:10.1186/s40478-019-0816-1)
Supplement: Supplementary file 8 — Additional file 8. Behavioural results. [file 40478_2019_816_MOESM8_ESM.docx]

**Additional file 8**

*Behavioural results:*

**CatWalk**

*Hind paw swing*: Mixed ANOVA detected no statistically significant interaction between the TDP-43^M337V^ expression and DOX reduction time on hind paw swing (the duration of time the paw was not in contact with the glass plate), n≥6/group, (F(_3.3, 76_) = 1.662, *p=*0.178; Supplemental Figure 3A) and no effect of genotype (F(_1, 23_) = 1.993, *p=*0.171). However, there was a significant effect of time (F(_3.3, 76_) = 4.289, *p=*0.006) suggesting that both groups took less time with hind paw swing over time.

*Hind paw swing speed*: There was no statistically significant interaction between the TDP-43^M337V^ and DOX reduction time on hind paw swing speed (speed of the paw while taking a step), however we were approaching significance, n≥6/group, (F(_4, 84_) = 2.374, *p=*0.059; Supplemental Figure 3B), and no main effect of genotype (F(_1, 21_) = 1.606, *p=*0.219). ANOVA found a main effect of time (F(_4, 84_) = 4.416, *p=*0.003), matching the hind paw swing time results indicating that both groups generally moved their hind paws faster over time. A potential training effect may be a possible explanation of these findings due to the high frequency of the CatWalk tests.

**Prepulse inhibition of the acoustic startle response**

*Baseline startle:* Mixed ANOVA detected no significant interaction between genotype (TDP-43^M337V^) and DOX reduction over time on mean baseline startle, n≥6/group, (F(_1.7, 37.1_) = 0.213, *p=*0.772; Supplemental Figure 4A). The main effect of time showed no significant difference in mean baseline startle at the different time points (F(_1.7, 37.1_) = 0.360, *p=*0.664). There was no statistically significant difference in mean baseline startle between ChAT-tTA/TRE-TDP-43^M337V^ and TDP-43 control (F(_1, 22_) = 0.860, *p=*0.364).

Within ChAT-tTA/TRE-TDP-43^M337V^ rats there was no significant interaction between tau and time on baseline startle response (n≥6/group, F(_4, 30_) = 0.807, *p=*0.097; Supplemental Figure 4B). The main effect of time showed no statistically-significant difference in mean baseline startle at the different time points (F(_2, 30_) = 0.020, *p=*0.980). There was no statistically significant difference in mean baseline startle between injection groups (F(_2, 15_) = 0.300, *p=*0.745).

*Prepulse inhibition (PPI)*: There was no significant main interaction detected by mixed ANOVA of TDP-43^M337V^ and time on PPI, n≥6/group, (F(_2, 48_) = 0.142, *p=*0.868; Supplemental Figure 4C). The main effect of time showed no significant difference in mean PPI at any time point (F(_2, 48_) = 0.142, *p=*0.263). There was a significant difference in mean PPI between ChAT-tTA/TRE-TDP-43^M337V^ and TDP-43 control, (F(_1, 24_) = 4.403, *p=*0.047), where ChAT-tTA/TRE-TDP-43^M337V^ animals generally had lower levels of PPI than the control group, regardless of the time point.

Within ChAT-tTA/TRE-TDP-43^M337V^ rats there was no significant main interaction of AAV9 vector injection group and time on PPI, n≥5/group, (F(_4, 34_) = 0.686, *p=*0.607; Supplemental Figure 4D). The main effect of time showed no statistically significant difference in mean PPI at the different time points, (F(_2, 34_) = 1.179, *p=*0.065). There was no statistically significant difference in mean PPI between injection groups, (F(_2, 17_) = 1.242, *p=*0.314).

**Open field testing**

*Total distance traveled:* Mixed ANOVA found no statistically significant interaction between TDP-43^M337V^) and DOX reduction time on cumulative distance traveled, (n≥5/group, (F(_1.6, 20.2_) = 2.057, *p=*0.161; Supplemental Figure 5A). The main effect of time showed no statistically significant difference in mean cumulative distance traveled at the different time points, (F(_1.6, 20.2_) = 0.836, *p=*0.421). There was no statistically significant difference in mean cumulative distance traveled between ChAT-tTA/TRE-TDP-43^M337V^ and TDP-43 controls (F(_1, 13_) = 0.478, *p=*0.502).

Within ChAT-tTA/TRE-TDP-43^M337V^ rats, there was no significant main effect detected by ANOVA of the interaction between the time and tau on cumulative distance traveled, n≥3/group, (F(_1.5, 10.5_) = 1.304, *p=*0.300; Supplemental Figure 5B). The main effect of time showed no statistically significant difference in mean cumulative distance traveled at the different time points (F(_3, 10.5_) = 0.302, *p=*0.824). There was also no statistically significant difference in mean cumulative distance traveled between tau injection groups (F(_2, 7_) = 3.568, *p=*0.085).

*Thigmotaxis*: Mixed ANOVA revealed a significant interaction between TDP-43^M337V^ and time on thigmotaxis, n≥4/group, (F_(2, 24_) = 4.495, *p=*0.022; Supplemental Figure 5C). The main effect of time also showed a statistically significant difference in mean thigmotaxis at the different time points, (F(_1, 12_) = 5.373, *p=*0.039), however *post hoc* t-tests with Bonferroni correction revealed no significant differences between the any of the time points (p *≥* 0.05). There was also a significant difference in mean thigmotaxis between ChAT-tTA/TRE-TDP-43^M337V^ and TDP-43 controls (F(_1, 12_) = 5.373, *p=*0.039), regardless of the time, suggesting that control animals generally spent more time in the perimeter as opposed to the centre of the open-field. Difference score = time spent in perimeter – time spent in centre.

There was no significant interaction between tau and time on thigmotaxis, n≥3/group, (F(_4, 14_) = 1.558, *p=*0.240; Supplemental Figure 5D). Furthermore, the main effect of time showed a statistically significant difference in mean thigmotaxis at the different time points (F(_2, 14_) = 10.813, *p=*0.001). *Post hoc* t-tests with Bonferroni correction revealed a significant difference between baseline (before DOX reduction) and 2 weeks following DOX reduction (*p=*0.004), regardless of AAV9 vector injection group, suggesting that over time animals spent significantly more time in the perimeter as opposed to the centre of the open-field (all other *post hoc* analyses were insignificant (*p≥*0.05). There was no statistically significant difference in mean thigmotaxis between injection groups (F (_2, 7_) = 1.104, *p=*0.383).

**Sociability and social recognition**

*Sociability:* Mixed ANOVA found no statistically significant interaction between TDP-43^M337V^ expression and time on sociability difference score, n≥3/group, (F(_2, 14_) = 0.943, *p=*0.413; Supplemental Figure 6A). The main effect of time showed no statistically significant difference in sociability difference score at the different time points, (F(_2, 14_) = 0.907, *p=* 0.426), and there was no statistically significant difference in mean sociability difference score between ChAT-tTA/TRE-TDP-43^M337V^ and TDP-43 controls (F(_1, 7_) = 0.574, *p=*0.473). Difference score = time spent with cage mate – time spent with empty.

No statistically significant interaction was observed between tau and DOX reduction time on sociability difference score, n≥2/group, (F(_4, 10_) = 2.584, *p=*0.102; Supplemental Figure 6B). The main effect of time showed no statistically significant difference in mean sociability difference score at the different time points (F(_2, 10_) = 0.287, *p=*0.757). There was also no statistically significant difference in mean sociability difference score between injection groups (F(_2, 5_) = 0.813, *p=*0.495).

*Social recognition and preference*: No statistically significant interaction between TDP-43^M337V^ expression and time on social preference difference score, n≥6/group, (F(_2, 28_) = 2.493, *p=*0.101; Supplemental Figure 6C). The main effect of time showed no statistically significant difference in social preference difference score at the different time points (F(_2, 28_) = 1.540, *p=*0.232). There was also no statistically significant difference in mean social preference difference score between ChAT-tTA/TRE-TDP-43^M337V^ and TDP-43 controls (F(_1, 14_) = 2.166, *p=*0.163). Difference score = time spent with novel rat – time spent with cage mate.

No statistically significant interaction between the AAV9 injection group and doxycycline reduction time on social preference difference score, n≥2/group, (F(_4, 16_) = 0.813, *p=*0.535; Supplemental Figure 6D). The main effect of time showed no statistically significant difference in mean social preference difference score at the different time points (F(_2, 16_) = 0.296, *p=*0.296). There was also no statistically significant difference in mean social preference difference score between injection groups (F(_2, 8_) = 1.429, *p=*0.295).
